# Noninvasive Monitoring of Polycarbonate Mechanochemical Properties under Broad-Band Photodegradation

**DOI:** 10.1021/acspolymersau.5c00198

**Published:** 2026-04-09

**Authors:** Irene Bargagli, Sara Mattana, Martina Alunni Cardinali, Laura Cartechini, Brenda Doherty, Aldo Romani, Lucia Comez, Marisa Pamplona, Marco Paolantoni, Daniele Fioretto, Rafaela Trevisan, Alessandra Vannini, Massimo Lazzari, Costanza Miliani, Paola Sassi, Francesca Rosi

**Affiliations:** † Istituto di Scienze e Tecnologie Chimiche “Giulio Natta” (SCITEC), CNR, 06123 Perugia, Italy; ‡ Department of Chemistry, Biology, and Biotechnology, Università degli Studi di Perugia, 06123 Perugia, Italy; § Istituto Officina dei Materiali (IOM), CNR, 06123 Perugia, Italy; ∥ Conservation Science Department, Deutsches Museum, 80538 München, Germany; ⊥ Department of Physics and Geology, Università degli Studi di Perugia, 06123 Perugia, Italy; # Conservazione e Restauro, Triennale Milano, 20121 Milano, Italy; ∇ Departamento de Química Física, Facultade de Química, and Centro Singular de Investigación en Química Biolóxica e Materiais Moleculares (CIQUS), Universidade de Santiago de Compostela, 15782 Santiago de Compostela, Spain; ○ Istituto di Scienze del Patrimonio (ISPC), CNR, 80134 Napoli, Italy

**Keywords:** Polycarbonate photodegradation, external reflection IR spectroscopy, Brillouin light scattering, noninvasive, preventive conservation, spectral markers, mechanochemical changes

## Abstract

A noninvasive vibrational spectroscopy approach is proposed to monitor the conservation state of historical polycarbonate (PC) design and heritage objects, with the aim of identifying early signs of degradation essential for effective preservation. Systematic investigations on reference PC samples, artificially aged under indoor and outdoor lighting conditions, using IR external reflection spectroscopy, enabled the noninvasive tracking of both photo-Fries rearrangement and photo-oxidation, the two well-known degradation pathways of PC. Since mechanical loss is a key issue in plastics, the correlation between chemical and mechanical properties was further investigated by combining IR spectroscopy with Brillouin light scattering (BLS). This correlative analysis provided a novel noninvasive analytical tool for monitoring the mechanochemical changes underlying yellowing and embrittlement, linking PC molecular degradation to its altered viscoelastic behavior. Although originally designed for use in cultural heritage studies, the proposed approach establishes a framework for evaluating and monitoring a wide range of polymeric materials across diverse industrial applications. By bridging the gap between fundamental preventive conservation and polymer science, it supports strategies aimed at safeguarding both cultural heritage and modern technological materials.

## Graphical abstract

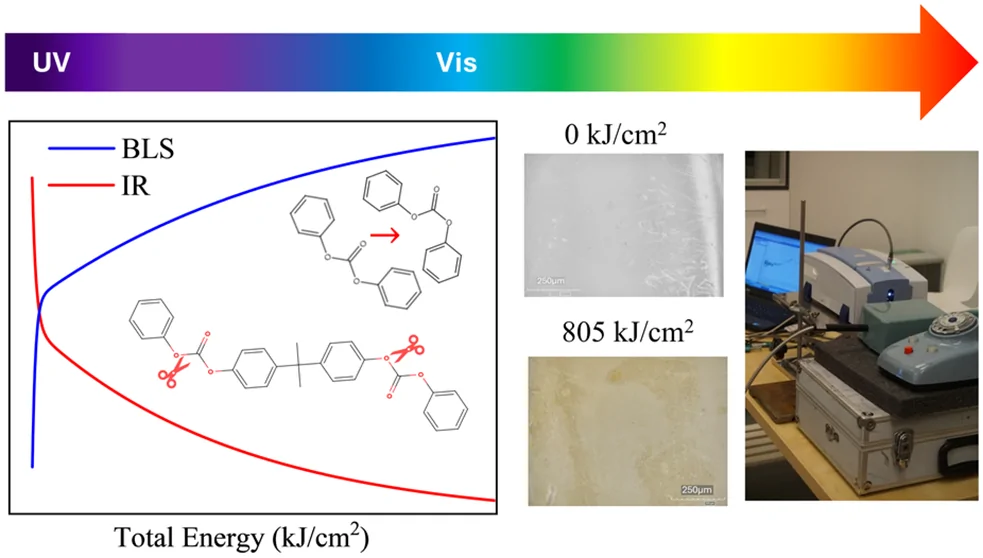

## 1 Introduction

Polycarbonate (PC) had a profound impact on modern engineering and design due to its exceptional mechanical strength, transparency, and versatility. The unique combination of these characteristics makes it widely used in a variety of applications (electronics, construction materials, medical applications, automotive, among others).^1^ It is also favored in the design of museum artifacts and increasingly employed in the creation of iconic architectural marvels, valued for its transparency, flexibility, lightness, and sustainability.^2^

Most commercial polycarbonates, marketed under trade names such as Lexan and Makrolon, are synthesized from bisphenol A (BPA) as the primary monomer. The resulting polymer consists of repeating units of aromatic rings connected by a central isopropylidene group (−C­(CH_3_)_2_–) and linked through carbonate groups (−O–C­(=O)–O−) (Figure 1). The diphenyl carbonate group of a BPA-PC chain shows trans–trans (t-t) and cis–trans (c-t) conformers through the rotation of the phenyl group around the carbonyl group (Figure 1b). These two conformations of BPA-PC reflect on its supramolecular structure:^3^ the crystalline phase features the trans–trans configuration, while both trans–trans and cis–trans coexist in the amorphous form, with t-t being the energetically favored state.^4–6^ This conformational distribution also depends on the production technique^6^ of the BPA-PC polymer and may evolve with aging as a consequence of the thermal history of the material.^5^

**Figure 1:**
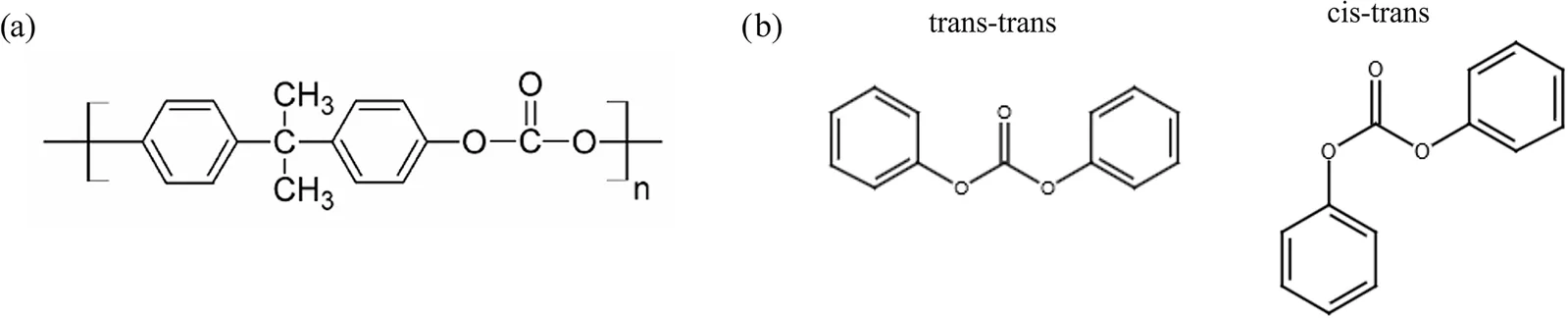
(a) Structural formula of the monomer of bisphenol A polycarbonate (BPA-PC); (b) trans–trans and cis–trans conformations of the diphenyl carbonate.

Typical effects of polycarbonate degradation exposed to light, humidity, and temperature are related to loss of transparency and discoloration, embrittlement and loss of toughness.^7^

Photodegradation is the main cause of alteration of BPA-PC optical and mechanical properties. It proceeds through two wavelength-dependent mechanisms:^8–11^ photo-Fries rearrangement and photo-oxidation. Both processes lead to the formation of photoproducts and structural changes that substantially impact the material performances.^12,13^

The photo-Fries rearrangement is triggered by UV light with wavelengths shorter than 300–330 nm. This mechanism involves the cleavage of C–O bonds in phenyl ester groups, resulting in the formation of reactive radicals. These radicals recombine to produce intermediates such as phenyl salicylate and dihydroxybenzophenone.^7,12–15^ Additionally, competing processes such as decarbonylation and decarboxylation may occur, leading to the formation of a complex mixture of species.^15^

Photo-oxidation becomes predominant at wavelengths longer than 310 nm. This mechanism begins with the abstraction of hydrogen atoms from the polymer’s methyl side chains, followed by isomerization. Primary radicals convert through chain-transfer-to-polymer transformations into tertiary radicals,^16^ which react with oxygen to form unstable hydroperoxides. These decompose into alkoxyl radicals, whose β-scission generates oxidation products such as ketones, alcohols, and acids.^9,12,17,18^ Moreover, other molecular changes induced by the photodegradation, such as cross-link and chain scission, can significantly affect PC’s mechanical properties.^19,20^

The aim of this study is to optimize a noninvasive in situ approach for the early detection of BPA-PC degradation, particularly in cultural heritage and design objects, as an essential step for their preservation. External reflection infrared spectroscopy was applied for detecting the chemical modification induced in BPA-PC due to photoaging. Both accelerated indoor (λ = 320–800 nm) and outdoor (λ = 295–800 nm) light aging were used to investigate photodegradation behavior enabling comparative trends under controlled conditions for BPA-PC either in museums or in exterior conserved architectural objects.

Attenuated total reflection of infrared radiation (ATR-IR) is the most widely applied modality of infrared spectroscopy for the study of polymers, including BPA-PC. However, within the domain of heritage science, noncontact and noninvasive approaches are normally preferred, or even constitute the sole viable option when fragile or delicate surfaces cannot tolerate physical contact.^21,22^ Among noninvasive methods, external reflection IR spectroscopy has been demonstrated to be a powerful tool to identify plastics of interest in design objects and to assess their conservation state through a noncontact approach.^22–26^

Driven by the need to optimize a noninvasive analytical tool for detecting early stage degradation, spectral markers observed in external reflection mode associated with both photo-Fries and photo-oxidation mechanisms were identified by leveraging the technique’s sensitivity to surface and subsurface properties.^22,27^ External reflection IR spectra were validated by comparison with ATR-IR profiles, thus serving as a benchmark to ensure the reliability of the spectral elaboration of the noninvasive spectroscopic data and associated findings.

The IR spectroscopy approach was complemented with Brillouin light scattering (BLS) spectroscopy^22^ to noninvasively probe the mechanical variations induced by aging and to elucidate how these changes relate to photoaging, chemical alterations, and conformational changes.^14,28–30^ Indeed, investigating this correlation is essential in degradation studies, particularly for plastics, where chemical changes often result in mechanical failures such as cracking or fracture, ultimately compromising material integrity and functionality.

While the primary objective of this work was to establish a method for monitoring, the results obtained from its systematic application to both indoor and outdoor aged samples also provided new insights into wavelength dependence of BPA-PC photodegradation. Indeed, although widely studied, the relationship between the two processes and the external factors triggering one over the other is still debated.^31^

Finally, the optimized noninvasive methodology was applied to BPA-PC objects from the cultural heritage collections of the Deutsches Museum and the Triennale Milano. For the latter, repeated measurements performed in 2016 and 2025 confirmed the suitability of the method as a long-term monitoring tool under real museum conditions. The study demonstrated the strength of IR characterization, which not only confirmed the plastic composition—consistent with findings from previous studies^21,25,32^ —but also enabled the detection of different conformational distributions suggesting insights into the polymer’s production history and processing conditions.

## 2 Materials and Methods

### 2.1 BPA-PC Mock-Ups and Artificial Aging Protocols

Bisphenol-A Polycarbonate (BPA-PC) sheets were purchased from SABIC Innovative Plastics (LEXAN 9030TG) and subsequently cut into 2 cm × 2 cm square samples. The thickness of the sheets was 1.5 mm.

For the outdoor lighting, BPA-PC sheets were subjected to photoaging using a Suntest XLS+ chamber equipped with a 1700 W xenon lamp in the wavelength range of 295–800 nm. A glass filter with a cutoff at λ < 295 nm excludes the radiation more energetic than that of outdoor daylight exposure. The maximum temperature of the samples during irradiation was 30 °C, as maintained by a forced air circulation system, and the irradiance was 765 W/m^2^ for a total time of 122 days, resulting in a total energy of 805 kJ/cm^2^. The indoor aging was performed by using a Cermax filtered xenon lamp (300 W, λ > 320 nm), placing the samples at 50 cm from the light source. The temperature during the irradiation was ca. 30 °C and the irradiance was 1633 W/m^2^ for a total time of 47 days, resulting in a total energy of 663 kJ/cm^2^.

These exposures are intended to compare degradation trends rather than to directly replicate natural aging, as the material response depends on multiple interdependent factors, including cumulative dose, exposure duration, temperature, humidity, and other environmental variables.

In Figure S1 of the Supporting Information (SI), the lamp emission spectra of the indoor (a) and outdoor (b) light aging are reported and compared with the UV–VIS absorption spectrum of the unaged BPA-PC sample.

### 2.2 BPA-PC Objects from the Deutsches Museum and the Triennale Milano Collection

The studied objects were a selection of polycarbonate plastic artworks from the Deutsches Museum and the Triennale collection (Figure S2). Specifically, these included the Apple iBook produced in the early 2000s, the Cellidor phone-0288, produced in 1958, with both the blue casing and transparent dial made of polycarbonate, the Boalum lamp-0092 and Boalum lamp-0731, both produced in 1970.

### 2.3 Colorimetry

Color measurements were performed by a Konica–Minolta CM700D portable colorimeter, equipped with a pulsed xenon lamp emitting into the visible spectral range, a silicon photodiode array detector and an integrating sphere with a 40 mm internal diameter. The software interfaced with the instrumentation allowed conversion of the spectra (recorded in the 360–740 nm range with 10 nm spectral resolution) into CIE L*a*b* chromatic coordinates under D65 standard illuminant and a 10° observer angle.

Three measurements were collected for each sample, and with the averaged values of the chromatic coordinates L*, a*, and b*, the total color changes were calculated and expressed in terms of Δ*E*

1 $$\Delta E=\sqrt{{\left(\Delta L\right)}^{2}+{\left(\Delta a\right)}^{2}+{\left(\Delta b\right)}^{2}}$$

The uncertainty (δ) in ΔE*, ΔL*, Δa*, and Δb* was calculated using the error propagation theory.

### 2.4 3D Digital Microscope

The high-resolution 3D digital microscope Hirox RX-100 was used to capture surface images at magnification of 320× to observe morphological variations and color heterogeneities.

### 2.5 UV–VIS Spectrometer

UV–VIS absorption spectra were recorded on a UV/VIS/NIR spectrophotometer (V-570 JASCO, Tokyo, Japan) equipped with Spectra Manager V-500 software. The spectra were acquired in the 200–800 nm range, with a 2 nm slit width.

### 2.6 IR Spectroscopy

#### 2.6.1 Portable FT-IR System

Noncontact Fourier Transform InfraRed (FT-IR) spectra were recorded by the Bruker Optics α-R spectrophotometer equipped with a SiC Globar IR radiation source, a Michelson interferometer (RockSolid design, Bruker Optics), and a room temperature deuterated l-alanine doped triglycine sulfate detector. The external reflection optical layout is 22°/22°, collecting the IR radiation reflected from a surface located at about 1 cm distance and with a probed area of about 6 mm in diameter. The investigated area is visualized by the universal serial bus video camera. External reflection-mode spectra, expressed as pseudoabsorbance *A*′ = log­(1/*R*), with *R* = reflectance, were recorded in the range of 8000–400 cm^–1^ using a gold mirror plate for the measure of the background, with a spectral resolution of 4 cm^–1^ and acquiring 144 scans. Three measurements were performed at different points of the aged surface for each sample.

IR band intensities were determined by a three-step procedure using the OPUS routine for the reflectance spectrum analysis: (1) application of the Kramers-Krönig (KK) transformation to the entire spectral range (8000–400 cm^–1^); (2) application of the second derivative to the obtained absorbance profile; (3) vector normalization (spectral range: 2300–400 cm^–1^). This procedure allows us to derive the absorbance, highlight the band changes, avoiding baseline treatment, and eliminating the spectral offset.

#### 2.6.2 Bench-Top FT-IR Systems

ATR-FT-IR spectra were acquired using a Thermo Nicolet 6700 spectrometer (Thermo Fisher Scientific, USA) equipped with a Smart Endurance accessory featuring a single-reflection diamond crystal and an MCT detector, operating at 4 cm^–1^ resolution with 128 scans. ATR intensities were wavelength corrected to obtain a pseudoabsorbance profile using the OPUS software.

The BPA-PC photoaging study was preceded by an evaluation of the BPA-PC external reflection profiles in the mid-IR range, to validate both the spectral positions and intensities of the k-index profiles obtained from the KK-correction by comparison with ATR-IR measurements.

The external reflection IR spectra of BPA-PC reference samples shown in Figure 2 (black lines) are dominated by Fresnel-type reflections,^21,27^ which give rise mainly to derivative-like bands caused by the variations in the real part of the refractive index (*n*) as a function of wavelength, and Reststrahlen/inverted bands, associated with weaker and stronger oscillators, respectively.^27^ The corresponding *k*-index profiles and ATR-IR spectra show very similar spectral features (Figure 2, gray and red lines) for both the unaged and the most highly exposed sample, thus suggesting the effectiveness of KK transformation. A summary of infrared band positions (*k*-index) and assignments from literature is provided in Table S1.

**Figure 2:**
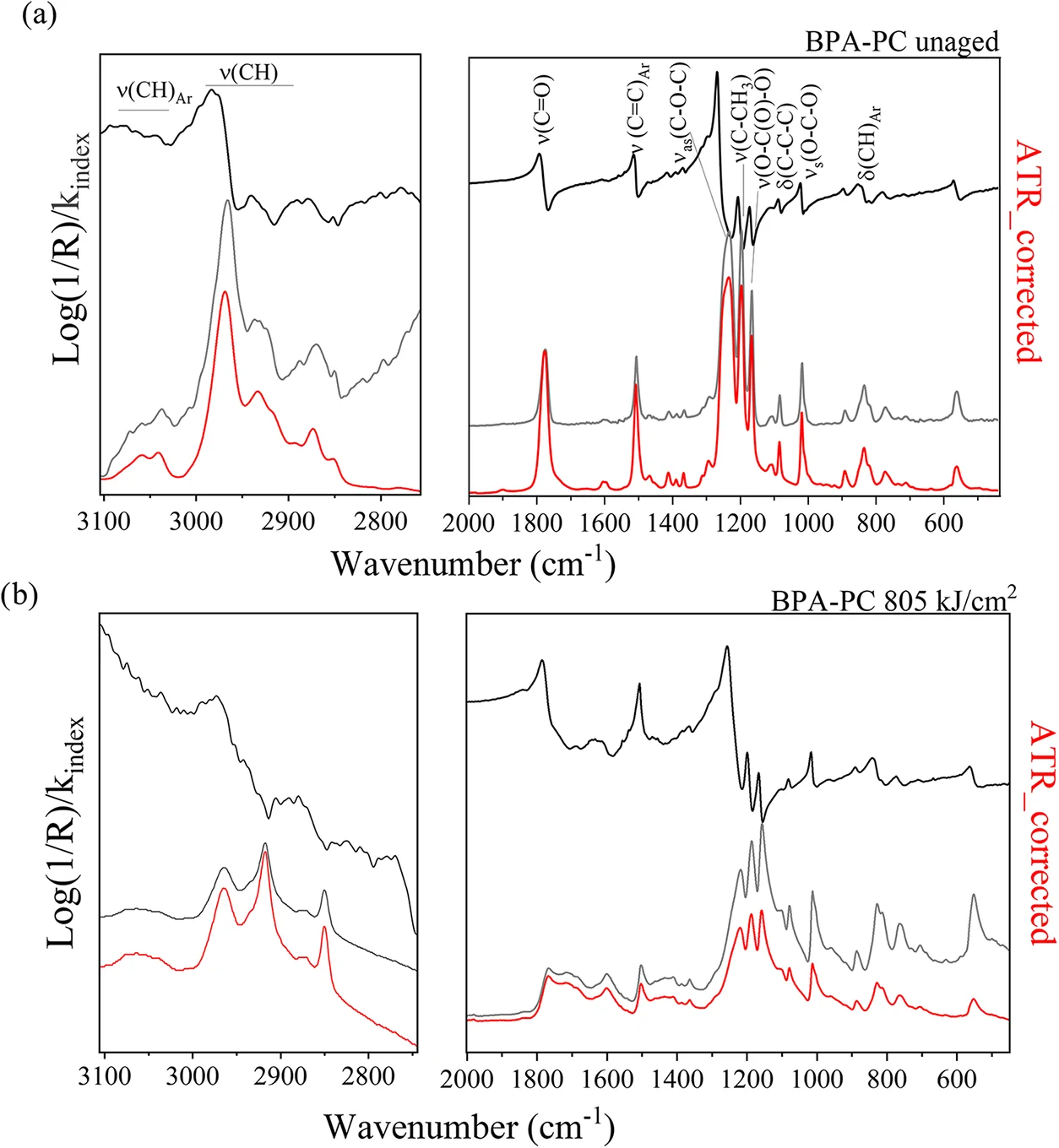
IR external reflection spectra (black lines) of the unaged (a) and the most highly exposed (b) BPA-PC samples. *k*-index profiles resulting from KK-correction (gray lines) and corresponding spectra acquired in ATR-IR mode (red lines). Main IR bands and corresponding assignments are also indicated. Ar = aromatic ring.

The IR experiments at variable optical layouts were performed with INVENIO FTIR Bruker spectrometer working in external reflection modality and using the variable angle specular reflectance accessory VeeMAX. II from PIKE Technologies. Spectra were recorded varying the incident angles from 30° to 80°, each spectrum being obtained at room temperature, averaging over 120 scans at a resolution of 4 cm^–1^. Measurements were performed in the 8000–400 cm^–1^ range and expressed as pseudoabsorbance *A*′ = log­(1/*R*). The penetration depths at each incidence angle were calculated according to eq 2.^33^

2 $$d_{p}=\frac{\lambda }{4\pi k}\sqrt{1-\frac{\sin^{2}\left(\alpha \right)}{n^{2}+k^{2}}}$$

where α is the incidence angle of IR radiation, λ the wavelength and *n* and *k* the optical parameters describing the complex refractive index, namely the real part *n* (refractive index) and imaginary part *k* (absorption index) taken from literature data.^34,35^

Where not explicitly stated, references to IR spectra in the text correspond to spectra acquired in external reflection mode.

### 2.7 Brillouin Light Scattering

Brillouin light scattering (BLS) relies on inelastic interactions between monochromatic light and thermally activated acoustic waves in the material, with frequencies in the gigahertz (GHz) range. This interaction causes a small frequency shift in the scattered light, called Brillouin shift, which reflects the local mechanical response through changes in acoustic wave velocity. As a result, BLS provides noncontact and nondestructive access to elastic and viscoelastic properties at small length scales.^22,36,37^ In the present study, BLS was employed to study the mechanical response of the aged and unaged BPA-PC samples. This information was correlated with the compositional changes monitored by IR spectroscopy.

A schematic illustration of the BLS setup used is reported in the Supporting Material (Figure S3). Experiments were performed using an incident laser radiation at 532 nm. The backscattered light was collected and analyzed using a high-resolution Sandercock-type tandem 3 + 3 pass Fabry–Pérot interferometer.^36^ The system is equipped with a confocal microscope, custom-designed to match the input of the interferometer and a motorized stage to allow sample mapping on the *xy* axes. Measurements were taken with the laser light focused on the sample surface using a 50× microscope objective, and the input and output pinholes of the interferometer were set to 70/200 to ensure confocality. This geometry allows for focusing the laser beam within a depth of 2.5 μm, as estimated by the axial resolution formula for confocal microscopy (eq 3)­

3 $$z\approx 1.4n\lambda /{\left(\mathrm{NA}\right)}^{2}$$

where *z* is the depth resolution, *n* = 1.57 ^38^ the refractive index, λ the laser source wavelength and NA = 0.55 the numerical aperture of the objective.

This configuration allowed us to determine the frequency position of the Brillouin doublets, which are related to the longitudinal elastic waves propagating in the medium. For each sample, three different points were probed using a mirror spacing of 6.5 mm and 450 scans.

## 3 Results

BPA-PC mock-ups were irradiated under both outdoor and indoor light conditions, being this thermoplastic polymer found both in museum objects and as exterior architectural elements and also taking into consideration the wavelength dependence of the polymer photodegradation.^8–10^ In Figure S1 the absorbance spectrum of unaged BPA-PC in the UV–VIS range is reported, showing that both the lighting conditions include wavelengths that are absorbed by the polymer.

The main text presents the spectroscopic study on the mock-ups artificially aged under outdoor lighting (λ = 295–800 nm) designed to promote and trigger both the photo-Fries rearrangement and photo-oxidation degradation pathways. The results of the indoor light aging are mainly reported in the SI and are referenced in the final section of the main paper, where the study of historical design museum objects made of BPA-PC is discussed.

### 3.1 Monitoring the Photodegradation of BPA-PC under Outdoor Light Conditions

Figure 3 shows the IR spectral modifications of the artificially photoaged BPA-PC samples involving both the carbonate, the aliphatic side chain groups, and the aromatic ring. The polymer carbonate moiety is identified by the derivative-like band associated with the C=O stretching^39,40^ at 1770 cm^–1^; the strong band at 1230 cm^–1^ associated with the ν­(C–O–C) antisymmetric stretching, incorporating the contribution from t-t and c-t conformers;^5,6^ and the band at 1160 cm^–1^ assigned to the ν­(O–C­(O)–O) antisymmetric stretching.^40,41^

**Figure 3:**
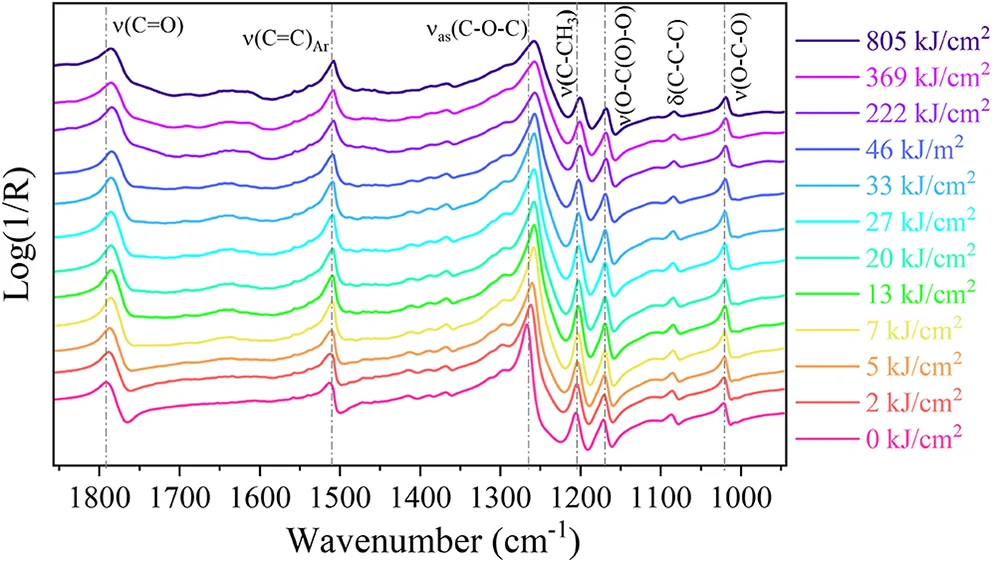
IR external reflection spectra of BPA-PC photoaged samples at increasing total energy (kJ/cm^2^).

The phenyl group is recognized by the ν­(C=C) stretching band of the aromatic ring at 1505 cm^–1^ ^39,40^ and the δ­(C–C–C) band of the para-substituted benzene ring at 1080 cm^–1^ of the aromatic component, both displaying derivative-like profiles.^40,42^ Finally, the polymer side chain is identified by the band at 1194 cm^–1^ associated with ν­(C–CH_3_) stretching in the gem-dimethyl position.^19,43^

The variations of these peaks can be used to monitor: (i) the newly formed photoproducts (with implications for polymer yellowing); (ii) the evolution of the photo-Fries and photo-oxidation pathways; and (iii) the polymer conformational changes.

#### 3.1.1 BPA-PC Yellowing and Formation of Degradation Products

Yellowing of the BPA-PC is one of the main issues associated with polymer aging, since colorlessness and transparency represent distinctive and essential properties for its wide range of applications, whether in design objects, in architectural contexts, or in numerous industrial applications. The effect of yellowing is evident in Figure 4a, where an image of the colorless and transparent unaged sample is compared with that of the sample irradiated at 805 kJ/cm^2^, showing clear yellowing. Figure 4b reports a closer view of the IR carbonyl spectral region (1900–1600 cm^–1^). This region is particularly significant for detecting and analyzing the formation of photoproducts associated with both photo-Fries rearrangement and oxidation.^8,12,13^ The almost derivative-like band at 1770 cm^–1^ is modified by the appearance of new bands associated with products of oxidative degradation (bands at 1720 cm^–1^ attributed to aliphatic chain acid dimers^7,11,12^ and at 1835 cm^–1^ to cyclic anhydrides,^12^ ★ marks in Figure 4b) and the photo-Fries rearrangement (bands at 1680 cm^–1^ and 1635 cm^–1^, assigned to phenylsalicylate and dihydroxybenzophenone^12,13^ respectively, ◆ marks in Figure 4b). ATR-IR spectra acquired on the same mock-ups (Figure S4) revealed that the newly formed photoproducts mainly contribute to the IR profiles as positive peaks (due to the volume reflection component of the light), only slightly shifted with respect to the ATR peak maxima.

**Figure 4:**
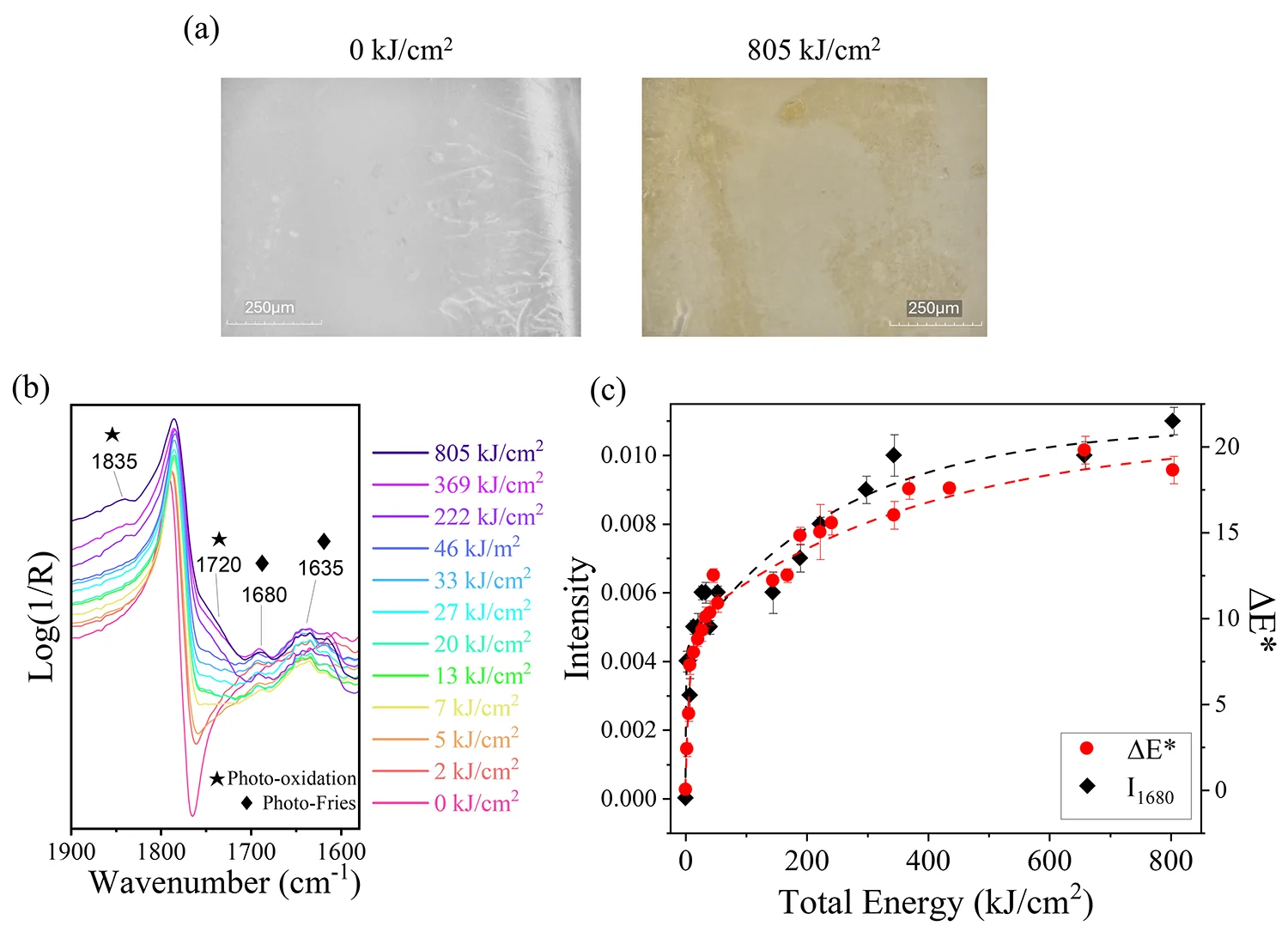
(a) Visible OM images of the (left) unaged and (right) the most exposed (805 kJ/cm^2^) BPA-PC samples; (b) IR spectra in the ν­(C=O) region, ★ and ◆ highlight the bands of the newly formed photoproducts associated with photo-oxidation and photo-Fries rearrangement, respectively; (c) color variation (expressed as ΔE*) and intensity variation of the band at 1680 cm^–1^ (associated with photo-Fries rearrangement) as a function of the total energy (kJ/cm^2^).

In Figure 4c, the band intensity at 1680 cm^–1^ attributed to the photo-Fries rearrangement is plotted against the imparted energy, highlighting a bimodal behavior with a very rapid and sharp increase in the formation of new products (before ca. 70 kJ/cm^2^), and a second stage with a slower generation of newly formed species. The evolution of the corresponding color change ΔE* (mainly represented by the variation of the b* parameter, indeed corresponding to yellowing, Table S2) is also shown. Both intensity at 1680 cm^–1^ and ΔE* variations as a function of total energy were fitted using biexponential functions (Figure 4c and Table S3) and showed very similar trends at low energies (<70 kJ/cm^2^), with closely matching *d*_1_ fit values.

This correspondence is particularly relevant for the early detection of BPA-PC photochemical degradation, which can be monitored using simple, rapid, and widely accessible colorimetry. Its integration with composition-sensitive methods, including IR spectroscopy, enhances the analytical capability of noninvasive in situ investigations.

#### 3.1.2 Photo-Fries and Photo-Oxidation Pathways

In the photo-Fries rearrangement, including the cleavage of the C–O bond of the phenyl ester group and the formation of two radicals, the carbonate moiety [ν_as_(C–O–C); ν­(O–C­(O)–O)] is directly involved (Figure 3). In the case of photooxidation, the side chain methyl groups are the primary targets for radical formation at the onset of degradation, which can be monitored by the ν­(C–CH_3_) band (Figure 3).^12,13,19^ The radical formation also correlates with structural changes in the polymer matrix, inducing the cleavage of the polymer backbone.^12,13^ Such scission likely results in the loss of para-substituted groups originally part of the polymer structure.

Figure 5 reports the intensity variation of the bands identified for monitoring the photodegradation as a function of the irradiated energy: the ν_as_(C–O–C) band at ca. 1230 cm^–1^, a marker of the photo-Fries rearrangement; the δ­(C–C–C) band of the para-substituted benzene ring at 1085 cm^–1^, and the ν­(C–CH_3_) band of the gem-dimethyl group at 1194 cm^–1^, both indicative of the photooxidative process.^42^

**Figure 5:**
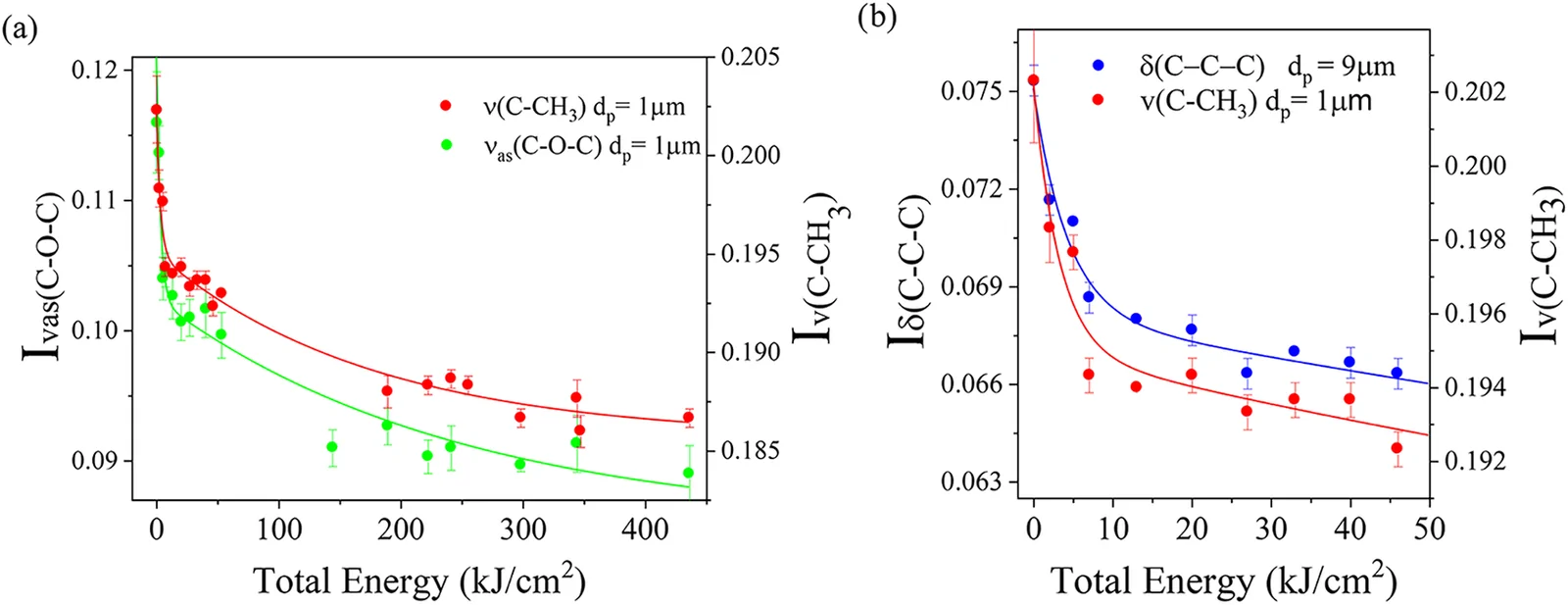
Intensity variation as a function of the total energy (kJ/cm^2^) for bands marker of polycarbonate photodegradation. (a) The ν_as_(C–O–C) at 1230 cm^–1^ and ν­(C–CH_3_) at 1194 cm^–1^ monitor surface changes at approximately 1 μm depth. (b) The ν­(C–CH_3_) at 1194 cm^–1^ and δ­(C–C–C) at 1085 cm^–1^ compare surface and subsurface levels during the photo-oxidation process. Estimated IR penetration depths (*d*_p_) for the three bands are also indicated.

Because photodegradation predominantly occurs at the surface,^13,44,45^ understanding the spectral changes in Figure 5 requires knowledge of the IR penetration depth (dp) associated with each monitored band. The calculated dp values, obtained using eq 2, are reported in Tables 1 and S1.

**Table 1:** Fitting Parameters of the Bi-Exponential Fitting Equations: *y* = *A*_1_ × exp­(−*x*/*d*_1_) + *A*_2_ × exp­(−*x*/*d*_2_) + *y*_0_, Best Describing the IR Intensity Changes of Selected Bands during Ageing^a^

band position	assignment	*d*_p_ (μm)	*d*_1_ (kJ/cm^2^)	*W*_A1_ (%)	*d*_2_ (kJ/cm^2^)	*W*_A2_ (%)	degradation process
1230 (cm^–1^)	ν_as_(C–O–C)	1	3.2 ± 0.9	53	244 ± 42	47	Photo-Fries
1194 (cm^–1^)	ν(C–CH_3_)	1	3.2 ± 0.8	44	177 ± 60	56	Photo-Oxidation
1085 (cm^–1^)	δ(C–C–C)	9	4.3 ± 0.7	28	404 ± 18	72	Photo-Oxidation
14.5 (GHz)	-	2.5>	2.5 ± 0.5	46	230 ± 57	54	-

a In the last row the best fitting of the BLS peak shift is also shown. For each signal, the derived constants (*d*_1_ and *d*_2_), reported as absolute values, their associated errors, and weight percentages (*W*_A1_% and *W*_A2_%) are reported.

Direct comparison of the ν­(C–CH_3_) and ν_as_(C–O–C) modes, both probed at approximately 1 μm depth, highlights that photo-oxidation and photo-Fries processes display a similar behavior. It is described by a biexponential function, showing a very rapid decrease within the first 20 kJ/cm^2^ (comparable *d*_1_ and relative weights, *W*_A1_, Figure 5a and Table 1) and a slower trend at longer exposure.

The influence of oxygen availability in BPA-PC photoageing is highlighted by comparing the behavior of two photo-oxidation markers that probe different material depths reflecting the varying penetration of IR frequencies (Figure 5b), and by examining the corresponding fitting results (Table 1). The ν­(C–CH_3_) band, associated with surface layers, exhibits faster and more pronounced chemical changes, whereas the δ­(C–C–C) band, which reflects a larger penetration depth, evolves more slowly due to the dependence on the diffusion rate of oxygen and reactive radicals.

Overall, the observed biexponential behavior, for the formation of degradation products and the associated yellowing, is consistent with the occurrence of two distinct degradation regimes. The low-energy component likely corresponds to primary radical-driven reactions, such as C–O bond cleavage and hydrogen abstraction. In contrast, the high-energy regime may include secondary processes like oxidation, cross-linking, or molecular rearrangements that stabilize degradation products. As these products progressively accumulate, oxygen diffusion into the polymer matrix may become rate-limiting.

From an analytical standpoint, it is noteworthy that with the portable IR system, operating in a 22°/22° optical layout, surface degradation products of BPA-PC can be detected across the mid-IR spectral range at depths of approximately 1 to 13 μm (Table S1). The capability to simultaneously monitor spectral markers of both photo-oxidation and photo-Fries rearrangements down to ∼1 μm is particularly significant for the early detection of BPA-PC degradation, with clear implications for preventive conservation.

#### 3.1.3 Conformational Changes

The analysis of the ν_as_(C–O–C) band at 1230 cm^–1^ also enables the evaluation of possible conformational changes of the BPA-PC mock-ups revealed by the evident band shift during aging, as shown in Figure 6a (BPA-PC unaged and 805 kJ/cm^2^ aged samples, red and blue lines, respectively). This band encompasses the spectroscopic contributions of both trans–trans and cis–trans configurations, which are more distinctly resolved in the second derivative spectrum, appearing at 1250 cm^–1^ and 1225 cm^–1^, respectively (Figure 6a,6b).^3^ The BPA-PC utilized in this study exhibits an amorphous structure characterized by both t-t and c-t conformers.

**Figure 6:**
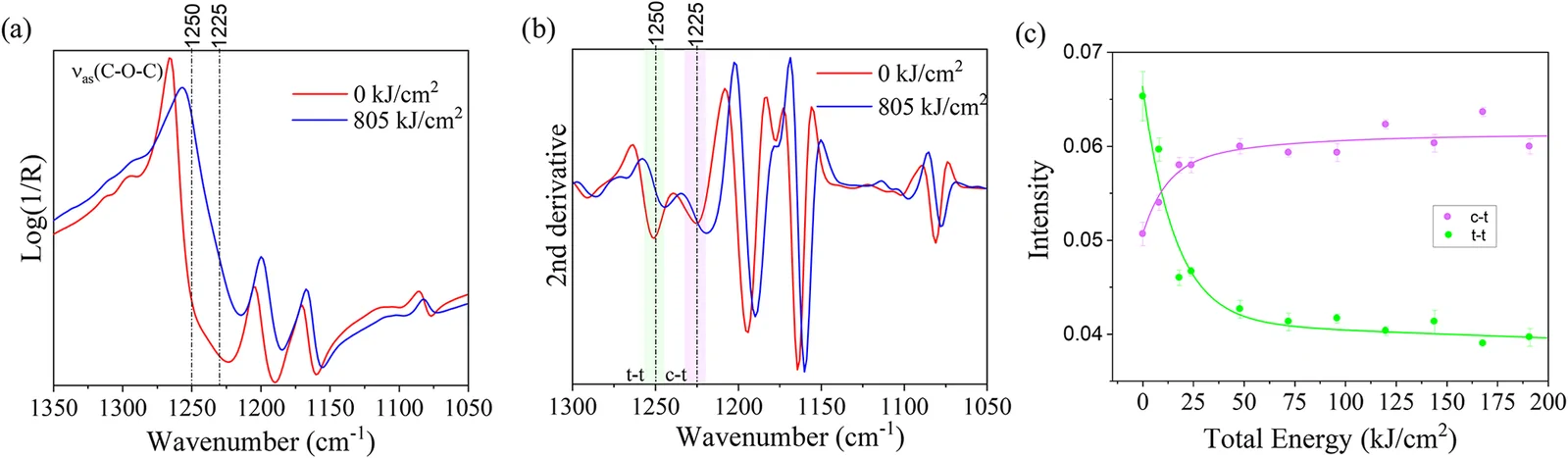
Comparison between an unaged BPA-PC sample (red line) and the 805 kJ/cm^2^ aged sample (blue line). (a) IR spectra in the range of the antisymmetric ν­(C–O–C) carbonate vibrational mode (1280–1220 cm^–1^); (b) second derivative spectra with the t-t and c-t conformer bands at 1250 (*I*_t‑t_) and 1225 (*I*_c‑t_) cm^–1^, respectively, highlighted; (c) the absolute value of the peak intensity of the t-t and c-t bands obtained from the second derivative plot is plotted as a function of the total energy.

Upon exposure to outdoor light aging, a change in the conformer population is observed (Figure 6c) with an increase in the c–t conformer leading to a shift of the t-t/c-t band intensity ratio, *I*_t‑t_/*I*_c‑t_, from 1.14 for the unaged sample to 0.42 for the most aged one (805 kJ/cm^2^). In literature, conformational rearrangements have been reported as a consequence of thermal treatments of BPA-PC.^3–6^

#### 3.1.4 Changes in Mechanical Properties

The IR spectral modifications were correlated with Brillouin data collected on the same samples offering an insight into the BPA-PC mechanochemical changes due to photodegradation. Figure 7a shows the Brillouin peak as a function of increasing total energy (kJ/cm^2^). The BLS frequency shift (ν_B_) moves toward higher frequencies, from 14.5 to 15.0 GHz, indicating a stiffening of the system (Figure 7b). By plotting ν_B_ along with the IR spectral variations representative of the photo-Fries arrangement [ν­(C–O–C) at 1230 cm^–1^] and photo-oxidation [ν­(C–CH_3_) at 1194 cm^–1^], a mirrored trend is clearly observed for both degradation pathways (dashed lines in Figure 7b). This behavior is confirmed by the fitting results reported in Table 1. The pronounced anticorrelation was further quantified using the Pearson correlation coefficient calculated from the linear fit of ν_B_ plotted against the intensity variation of the two vibrational modes. A very strong correlation (*R* = −0.99) was observed for the ν­(C–CH_3_) band (Figure 7c), while a slightly lower, yet still significant, correlation (*R* = −0.93) was found for the ν­(C–O–C) band (Figure 7d).

**Figure 7:**
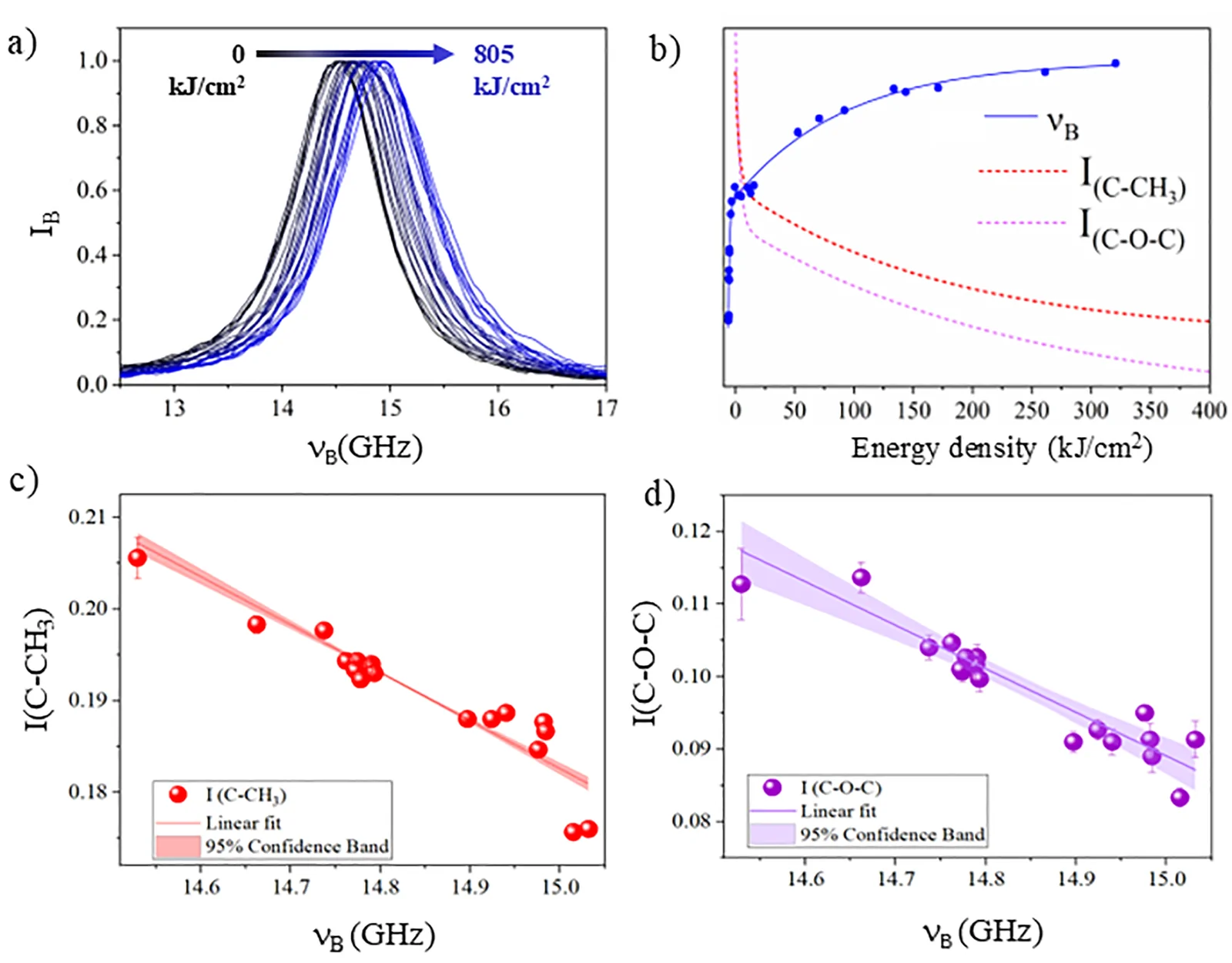
(a) Normalized Brillouin peak as a function of the total energy. (b) Brillouin frequency shift (ν_B_) versus total energy (kJ/cm^2^), fitted with the biexponential model (continuous line, Table 1). IR intensity trends of the 1194 and 1230 cm^–1^ bands, corresponding to the *I*(C–CH_3_) and *I*(C–O–C) vibrations, are also reported for comparison as dotted lines. Correlation plot of ν_B_ vs the intensity of the *I*(C–CH_3_) (c) and the *I*(C–O–C) bands (d).

These results suggest that the modification of the polymer’s mechanical properties is primarily influenced by the photo-oxidation mechanism. In particular, the observed stiffening of the material may be related to the formation of radicals at the CH_3_ groups which, according to the photo-oxidation mechanisms of BPA-PC reported in the literature,^12^ can initiate cross-linking reactions.^19,20,46^ This observation is consistent with the assumption that the radical formation may precede—or even contribute to—the suppression of specific BPA-PC molecular relaxation channels, such as the δ-relaxation associated with CH_3_ rotation.^44^

Our findings are also in line with those obtained by monitoring the variation in nanohardness of photoaged BPA-PC using atomic force microscopy nanoindentation.^19^ In that study, changes in elastic properties—quantified in terms of Young’s modulus—were correlated with a decrease in intensity of the ν­(C–CH_3_) band, and were attributed to the formation of a radical at the geminal dimethyl position, which not only initiates photo-oxidative reactions but also serves as the starting point for cross-linking processes. Although the literature primarily attributes the hardening of BPA-PC to photo-oxidation processes, the photo-Fries rearrangement may also contribute, as it similarly promotes cross-linking.

### 3.2 Comparison of the Indoor and Outdoor Photodegradation of BPA-PC

The IR spectral variations induced by indoor light aging (λ > 320 nm) are compared with those observed under outdoor exposition (λ > 295 nm) in Figure S5, indicating that no photo-Fries nor photooxidation takes place, in agreement with previous literature.^17,45^ Only a slight decrease in the t–t conformation relative to the c–t one, expressed by the intensity ratio *I*_t‑t_/*I*_c‑t_, can be observed, shifting from 1.14 in the unaged sample to 0.95 in the most exposed one (Figure 8a). The fact that conformational changes also occur under indoor conditions, albeit to a minor extent, corroborates that they result from a photoinduced rearrangement of the system^3–6^ rather than from radical reactions.

**Figure 8:**
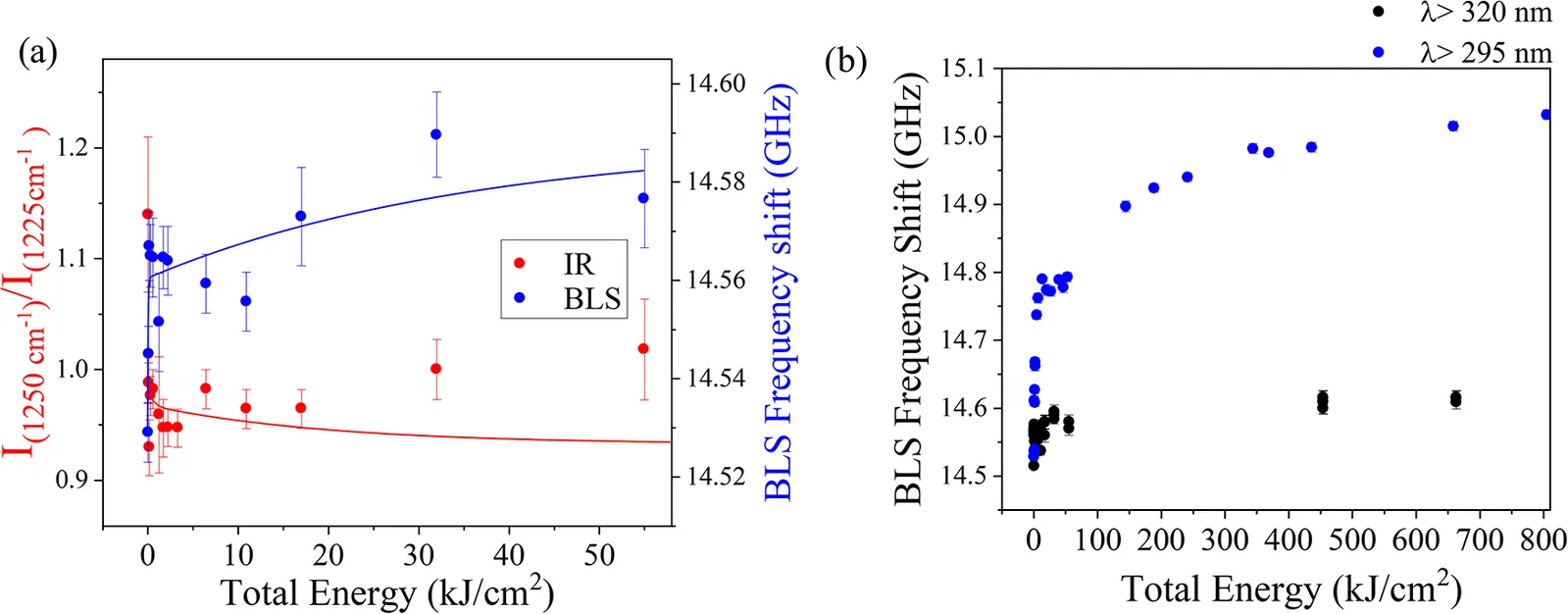
(a) IR and BLS data as a function of total energy for the indoor aging experiment: red circles refer to the intensity ratio of t-t and c-t conformer bands *I*_t‑t_/*I*_c‑t_ at 1250 and 1225 cm^–1^, respectively; blue circles refer to the BLS frequency shift. (b) BLS frequency shift measured for indoor- and outdoor-aged samples (black and blue circles, respectively).

Similarly, the mechanical properties of BPA-PC in the GHz range are only slightly affected by indoor exposure compared to outdoor conditions, exhibiting a modest increase in frequency shift of about 0.1 GHz (Figure 8b). This minor variation may be related to conformational transitions from t–t to c–t, consistent with the trend observed in the IR spectra (Figure 8a). According to Henrichs et al.,^47^ the shift of the carbonate group induced by the motion of the phenylene rings from the t-t to c-t conformation can promote a distortion of the carbonate units. This is likely to facilitate π-phenylene ring flipping, an effect that is associated in the literature with the characteristic γ-relaxation of BPA-PC.^48–50^ Indeed, this process, which involves multiple components, can couple with density fluctuations and thus affect the PC mechanical properties in the GHz regime. A further indication that indoor exposure affects the phenylene ring is provided by the slight decrease in the intensity of the ν­(C=C) IR absorption at 1505 cm^–1^ (Figure S5), which may be associated with the observed conformational change.

From the overall results, it can be inferred that aging under broad-spectrum irradiation including λ > 295 nm promotes the formation of highly reactive radicals whose concentration increases with accumulated dose, thereby enabling secondary processes such as oxidative cross-linking and molecular rearrangements. Such processes are directly reflected in the observed stiffening of the material (Figure 7b).^17,45^ In contrast, the spectral modifications observed under indoor-aging conditions (320–800 nm) reveal a different picture. Here, infrared and BLS data (Figure 8) point predominantly to conformational variations rather than radical-driven chemistry. This suggests that under lower-energy irradiation, degradation proceeds more subtly, through shifts in conformational equilibria (t–t versus c–t states) without significant cross-linking or oxidative hardening.

### 3.3 In Situ Noninvasive Monitoring of BPA-PC Design Objects under Indoor Light Exposure

The selected polycarbonate objects were sourced from prominent museums, including the Triennale Milano in Milan and the Deutsches Museum in Munich. The objects investigated are depicted in Figure S2 with also indicated the IR measuring points. External reflection IR spectra of the polycarbonate museum object, compared to the BPA-PC unaged and indoor photoaged samples, are reported in Figure 9.

**Figure 9:**
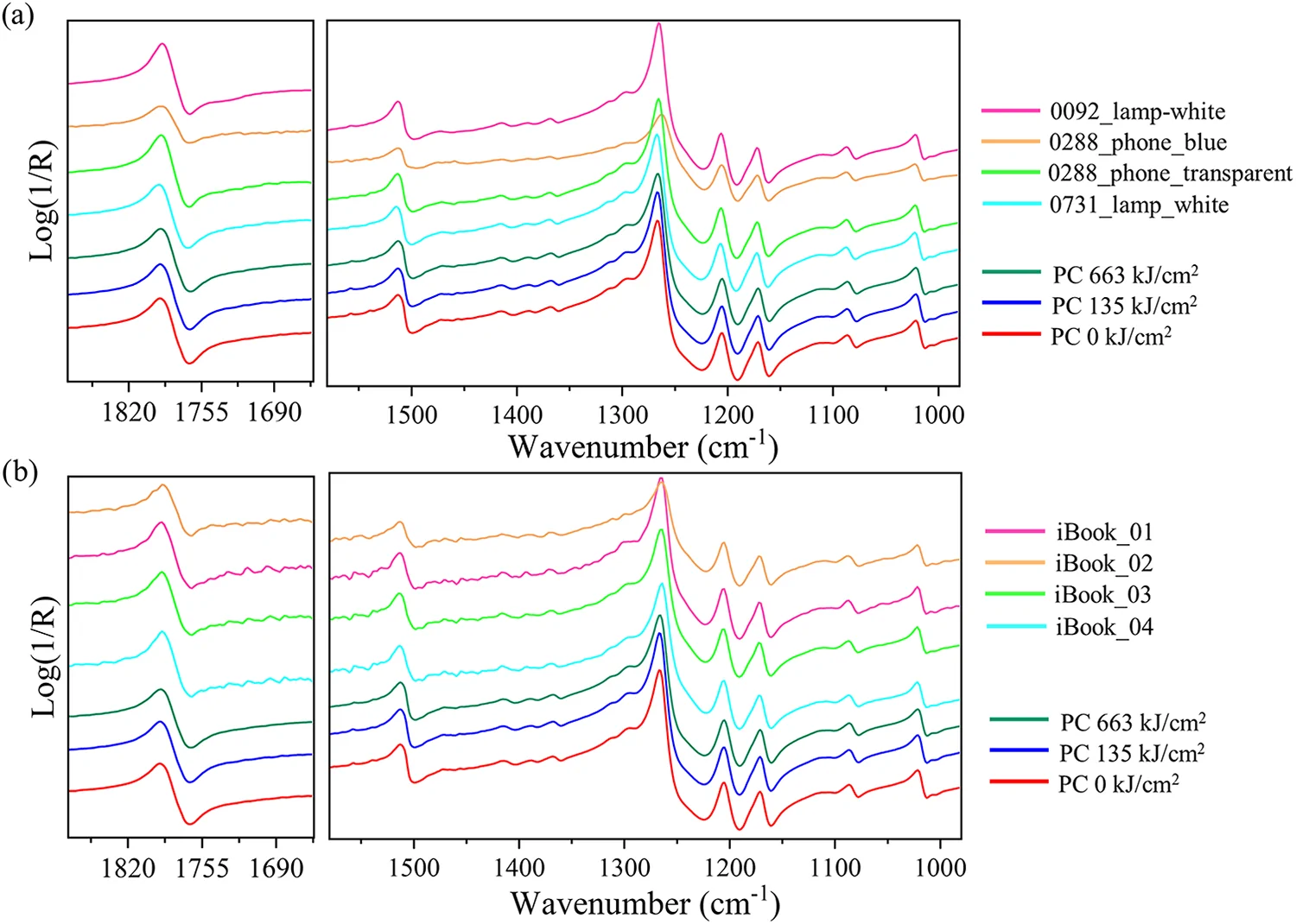
IR spectra of the BPA-PC museum objects compared with the reference samples unaged and photoaged in indoor condition at 0, 135, and 663 kJ/cm^2^. (a) Boalum lamp-0092, Boalum lamp-0731, and Cellidor phone-0288 (Triennale Design Museum) and (b) Apple iBook (Deutsches Museum).

All the investigated objects exhibit spectra that closely match the unaged polycarbonate reference, with no detectable bands of photoinduced degradation products in the 1900–1600 cm^–1^ range. These findings are consistent with the results obtained from indoor light aging on the BPA-PC reference samples, where no modifications associated with photo-Fries rearrangement or photo-oxidation were observed. On the other hand, most of the museum items consistently show a different relative abundance of the t-t and c-t conformers with higher *I*_t‑t_/*I*_c‑t_ compared to the two extreme reference cases (the unaged and the most exposed BPA-PC samples), which define a range of 0.95–1.14 according to our indoor photoaging model (Table 2). An exception to this trend is the blue case of the Cellidor phone which shows a shift of the 1230 cm^–1^ band (Figure 9a, orange line) corresponding to a *I*_t‑t_/*I*_c‑t_ ratio of 1.06 with respect to a *I*_t‑t_/*I*_c‑t_ of 1.46 for the transparent rotary dial.

**Table 2:**
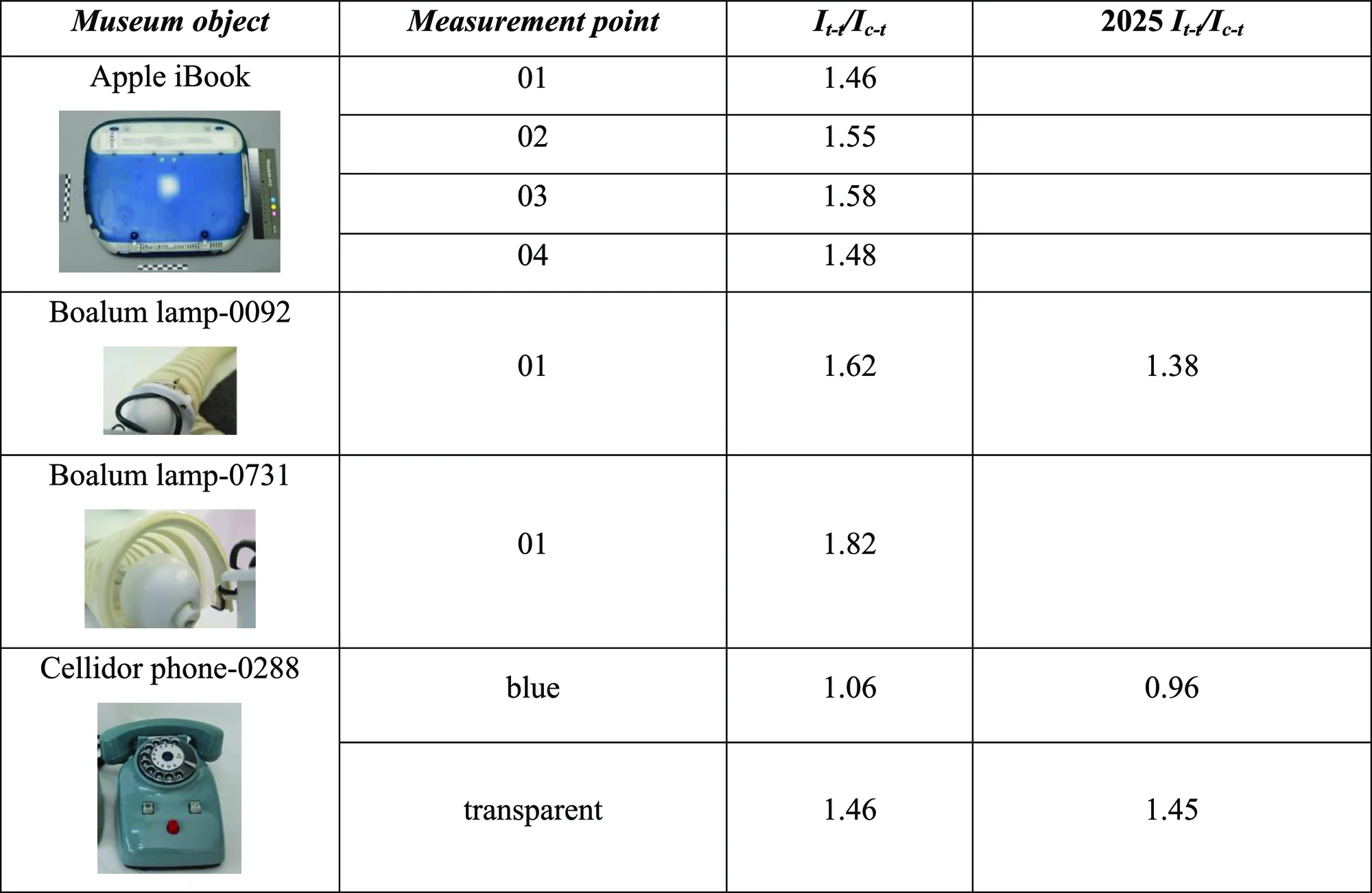
Museum Objects from the Deutsches Museum and from the Italian Triennale (Accessed in 2016 and 2025) Investigated by Non-Invasive IR External Reflection Spectroscopy and Corresponding Calculated *I*_t‑t_/*I*_c‑t_ Ratios

Variable-angle external reflection IR experiments were performed on the BPA-PC sample to investigate how the abundance of the two conformers varies with depth. As already discussed, the fraction of t-t structure diminishes with light exposure (Figure S6); besides, before aging, a surface layer, mainly composed of t–t conformers, extends from the outermost interface to a depth of ≈0.8 μm. The gradient distribution in the unaged sample may result from a nonuniform thermal treatment across its 1.5 mm thickness, with a more efficient annealing in the outer layers. This may have promoted the formation of the more stable t-t conformation^4^ in the external layer, which progressively decreases with depth. Indeed, annealing processes are applied during the plastic production to relieve internal stresses caused by extrusion or molding and to improve the mechanical and thermal properties.^51^ Since BPA–PC is an amorphous polymer, annealing at temperatures just below the glass transition (*T*_g_) are typically used to increase the t–t conformation and stabilize the material.^6^

Within this context, the conformational differences detected in the museum objects cannot be directly attributed to the conservation state of the objects (due to the lack of information about their initial condition) but rather to different manufacturing processes. A further variable affecting the conformational ratio can be related to composition. This is the case of the blue case of the Cellidor telephone where the lower *I*_t‑t_/*I*_c‑t_ ratio is most probably due to the presence of the ABS blended with PC as shown in Figure S7. In fact, it is reported that ABS diminishes the conversion to the t-t conformation during the annealing.^52^

Interestingly, new FTIR measurements performed in 2025 on the Boalum lamps and the Cellidor phone (Table 2) revealed a slight shift in the *I*_t‑t_/*I*_c‑t_ ratio in favor of the c-t conformer. These observations indicate that conformational rearrangement is an intrinsic property of the material that can occur over time even under controlled museum conditions. While single measurements cannot fully separate the effects of aging from those of composition or thermal history, repeated measurements, as conducted on selected objects from the Triennale collection, allow the use of conformational ratios as meaningful indicators for long-term monitoring of intrinsic material changes.

## 4 Conclusion

A multitechnique and multimodal approach, combining noninvasive infrared spectroscopy in ATR and external reflection modes with benchtop nondestructive BLS spectroscopy, enabled us to optimize a methodological approach to monitor noninvasively and in situ the mechanochemical degradation of BPA-PC associated with photo-Fries and photooxidation reactions. The study was based on the systematic investigation of laboratory samples aged under controlled indoor and outdoor irradiation conditions.

The interpretation model developed on laboratory samples exploits the capabilities of external reflection IR spectroscopy to detect extremely thin degradation layers in a noninvasive, noncontact manner using a portable device to work in situ. A reduced penetration depth of probing radiation is highly advantageous for studying surface phenomena, especially in materials like polycarbonate, where degradation is confined to the near-surface region.

The developed approach allowed us to monitor the primary degradation mechanisms of BPA–PC, namely photo-Fries rearrangement and photooxidation, which are triggered by irradiation at λ > 295 nm (outdoor aging) through the formation of highly reactive radicals. These primary reactions subsequently lead to secondary processes such as cross-linking and molecular rearrangements. The resulting degradation markers monitored by noncontact in situ IR spectroscopy, correlate with colorimetry, tracking yellowing, and BLS, which probes the progressive stiffening of the material.

Notably, with indoor photoaging (λ > 320 nm), photooxidation, though expected at these wavelengths,^9,10^ does not occur, suggesting that the process is primarily activated by radicals formed at higher photon energies. This is consistently confirmed by the analysis of real historical BPA-PC objects conserved indoor in museums, which similarly exhibit the absence of photooxidation signatures. This finding adds a new element to the understanding of the debated role of light energy dependence in both photo-Fries and photooxidation reactions of BPA-PC.^10,13^

A further finding of our study was the evidence that light exposure, similar to thermal annealing, affects the distribution of the c-t and t-t conformations in BPA-PC. These conformational changes are not wavelength dependent, and occur, to varying degrees, under both indoor and outdoor light aging. Light aging, in fact, promotes the less stable c-t conformer, more significantly under outdoor conditions, potentially compromising the chemical-physical properties and the overall stability of polycarbonate.

Finally, the study of historical museum objects made of BPA-PC by external reflection IR spectroscopy allowed us to compare their spectral behavior with that of the artificially aged reference materials. The noninvasive approach revealed a well-preserved state of the objects in agreement with the systematic study carried out on the indoor exposed reference samples. Indeed, no IR marker bands of photo-Fries rearrangement and photo-oxidation have been detected. Slight spectral changes were mainly attributed to different distributions of the t-t and c-t conformers linked to manufacturing and processing conditions as well as to specific blend compositions (i.e., the ABS-PC of the blue case of the Cellidor). Obviously, without knowledge of the initial condition of the studied objects, it is not possible to obtain a detailed picture of the potential transformation processes that occurred in the materials. However, these data establish a reference point for long-term monitoring, allowing observation of potential material changes over time, as demonstrated for two design objects measured in 2016 and revisited in 2025 at the Italian Triennale museum. Indeed, it will be of interest to further investigate whether the differences in polymer conformations could influence long-term stability of BPA-PC based materials.

Finally, although originally developed for the preventive conservation of synthetic polymers in historical objects, this approach has broader implications for polymer science and engineering. Chain scission and cross-linking are degradation pathways common to a wide range of polymer families and are typically coupled with mechanical changes. The proposed correlative approach enables the noninvasive detection of the earliest onset of mechanochemical deterioration before macroscopic failure occurs. This is significant in any application where polymeric components must maintain their performance over time, from cultural heritage materials to aerospace, automotive, and structural engineering.

## Supplementary Material



## Notes

Characteristics of the indoor and outdoor light aging (S1); images of the historical objects investigated (S2); micro-Brillouin light scattering setup (S3); ATR spectra of BPA-PC samples (S4); IR spectral variation of BPA-PC under indoor and outdoor light aging (S5); conformational ratio as a function of depth through the sample (S6); IR spectra of the Cellidor phone-0288 (S7); BPA-PC infrared bands and assignments (Table S1); colorimetry results (Table S2); fitting parameters for infrared spectral and color changes under outdoor light aging (Table S3) (PDF)

## ◆

Historic England, Fort Cumberland, Portsmouth PO4 9LD, UK
