# Supplementary material for: Noninvasive Monitoring of Polycarbonate Mechanochemical Properties under Broad-Band Photodegradation
Source: ACS Polym Au. 2026 Apr 9;6(5):1241–53. doi: 10.1021/acspolymersau.5c00198 (PMC13673879; doi:10.1021/acspolymersau.5c00198)
Supplement: Supplementary file 1 [file lg5c00198_si_001.pdf]

Supporting Information

Noninvasive Monitoring of Polycarbonate Mechanochemical Properties under Broad-Band Photodegradation

Irene Bargagli[a,b,†,*], Sara Mattana[a], Martina Alunni Cardinali[a,b], Laura Cartechini[a], Brenda Doherty[a], Aldo Romani[b], Lucia Comez[c], Marisa Pamplona[d], Marco Paolantoni[b], Daniele Fioretto[e,c], Rafaela Trevisan[f], Alessandra Vannini[f], Massimo Lazzari[g], Costanza Miliani[h], Paola Sassi[a,b], Francesca Rosi[a,*]

[a]Istituto di Scienze e Tecnologie Chimiche "Giulio Natta" (SCITEC), CNR, 06123 Perugia, Italy
[b]Department of Chemistry, Biology and Biotechnology, Università degli Studi di Perugia, 06123 Perugia, Italy
[c]Istituto Officina dei Materiali (IOM), CNR, 06123 Perugia, Italy
[d]Conservation Science Department, Deutsches Museum, 80538 München, Germany
[e]Department of Physics and Geology, Università degli Studi di Perugia, 06123 Perugia, Italy
[f] Conservazione e Restauro, Triennale Milano, 20121 Milano, Italy
[g]Departamento de Química Física, Facultade de Química, and Centro Singular de Investigación en Química Biolóxica e Materiais Moleculares (CIQUS), Universidade de Santiago de Compostela, 15782 Santiago de Compostela, Spain
[h] Istituto di Scienze del Patrimonio (ISPC), CNR, 80134 Napoli, Italy
[†]current address Historic England, Fort Cumberland, Portsmouth, PO4 9LD, UK

*corresponding authors: irene.bargagli@historicengland.org.uk; francesca.rosi@cnr.it;

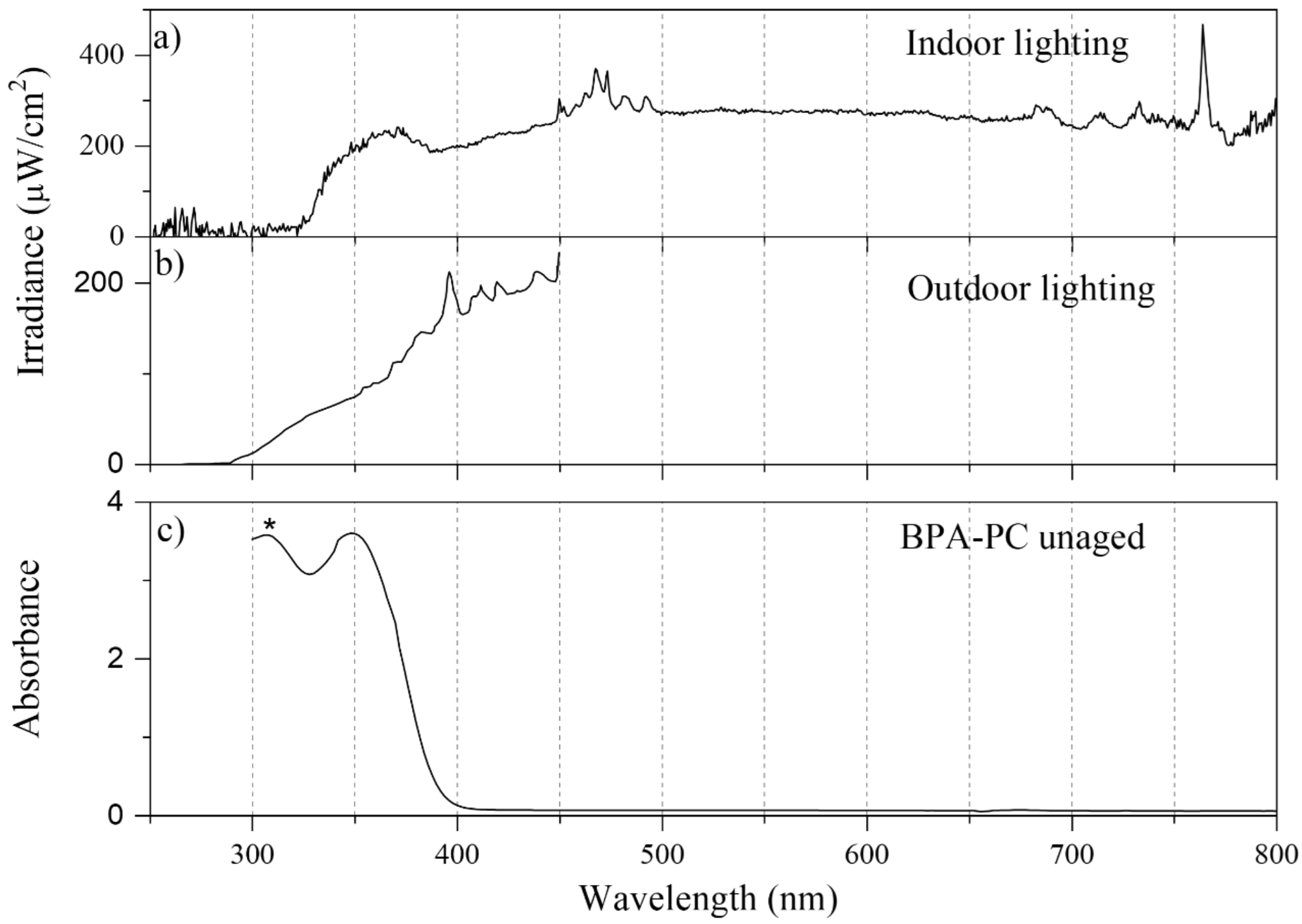

Figure S1*:* a) emission spectrum of the Cermax filtered xenon lamp for the indoor exposure (λ > 320 nm); b) emission spectrum of the Suntest XLS+ chamber lamp for the outdoor exposure (λ> 295 nm); c) UV-VIS absorbance spectrum of unaged BPA-PC sample. Due to saturation, the absorbance spectrum below 350 nm is affected by spectral distortions, and the marked peak is an artefact (*).

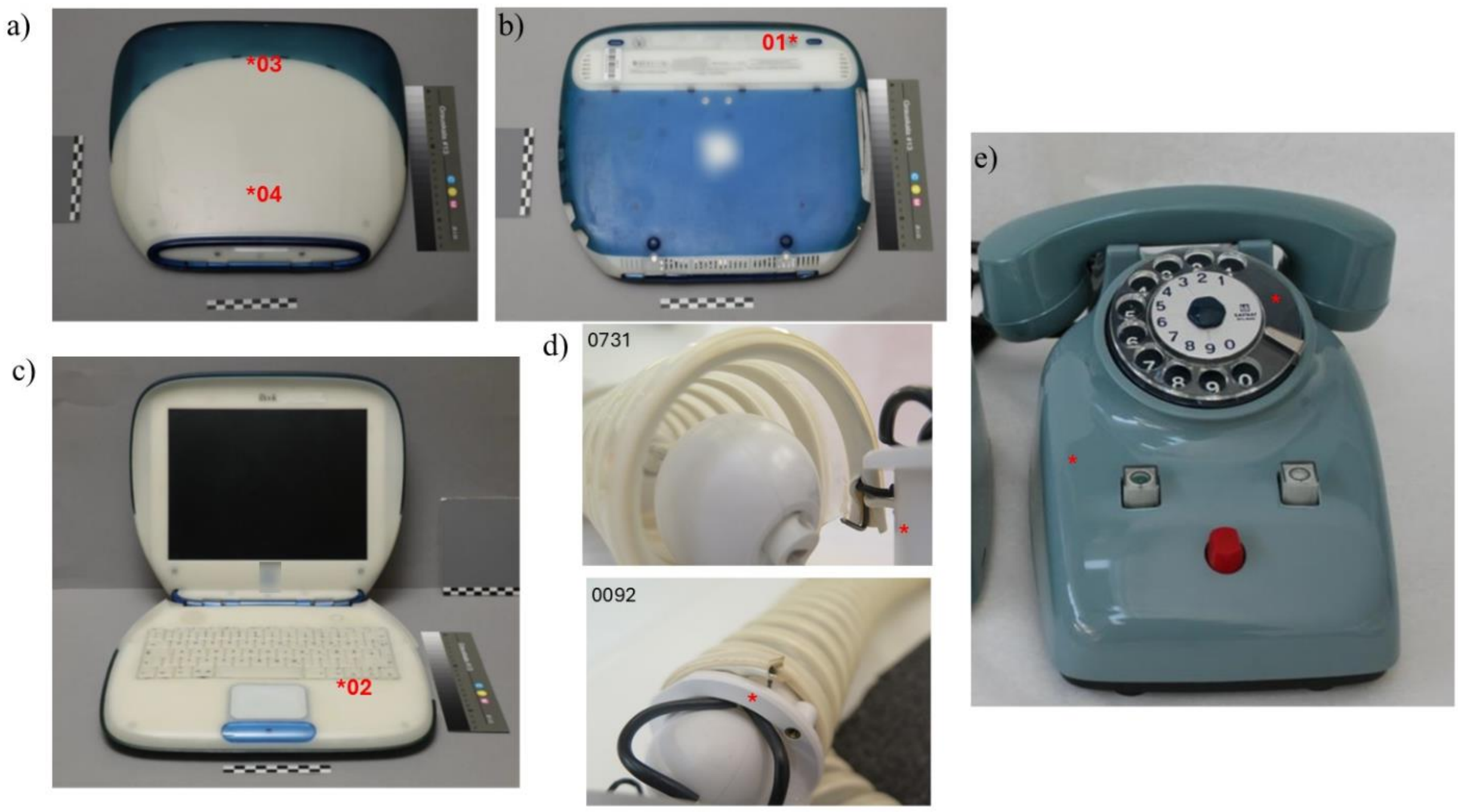

Figure S2: BPA-PC historical objects investigated in this study, (a–c) Apple iBook (Deutsches Museum); (d) Boalum lamp-0092 and Boalum lamp-0731; (e) Cellidor phone (Triennale Milano). Analysis points are highlighted in red.

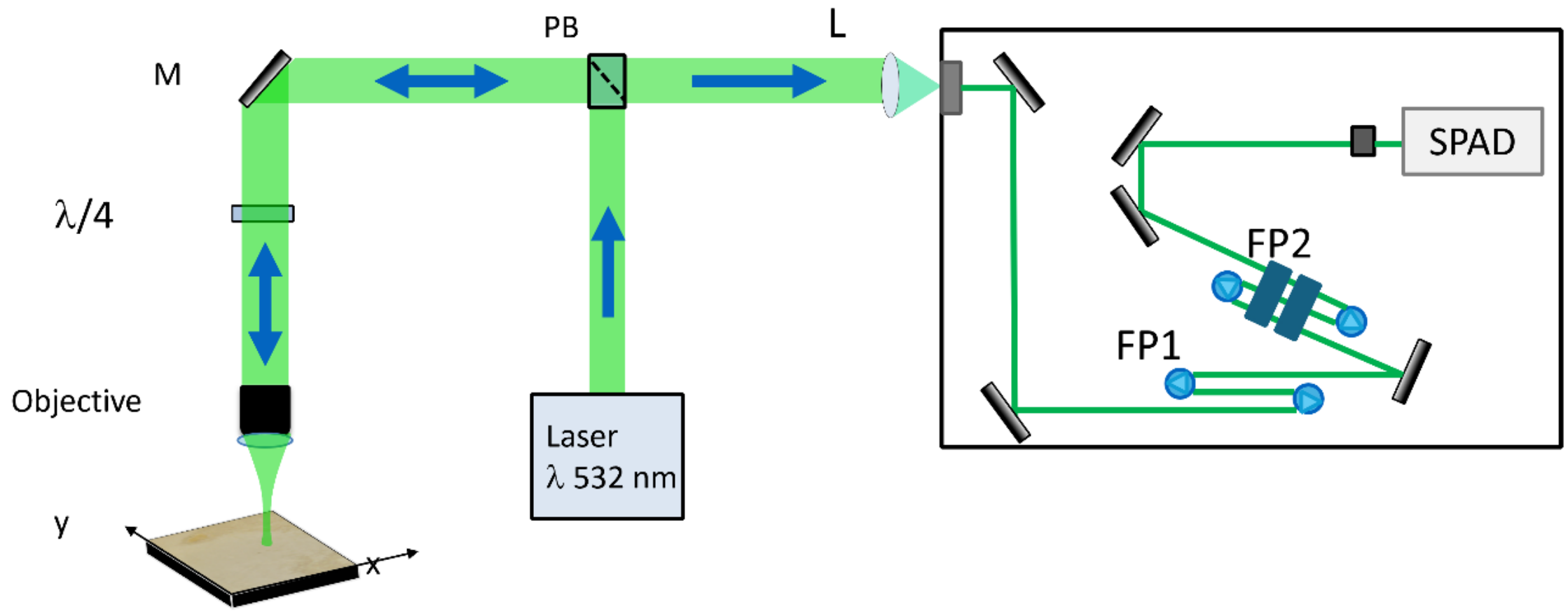

Figure S3: Schematic representation of the Brillouin light scattering (BLS) setup used for the experiments. A laser beam from a 532 nm single-mode solid-state laser is directed by a polarizing beam splitter (PB) and a mirror (M) and focused onto the sample through a microscope objective. The sample is mounted on an xyz microtranslation stage. The same objective collects the light backscattered from the sample; a λ/4 wave plate placed upstream of the objective allows switching between depolarized and unpolarized scattering configurations. After passing through the polarization optics and lens (L), the signal is sent to a tandem (3+3) pass Fabry-Perot interferometer system (FP1, FP2) and finally detected by a single-photon avalanche photodiode (SPAD).

Table S1: Polycarbonate (BPA-PC) infrared band maxima and assignments. The estimated penetration depth, considering an incidence angle value of 22°, has been calculated according to Equation (2).

Peak position (k-index) (cm^{-1})	Assignment	Estimated penetration depth (μm) (Eq. 2)[1]
558	*p*-substituted ring absorption[2]	
830	=CH out-of-plane deformation[2]	
1015	ν_s(O-C-O)[3]	5
1085	δ (C-C-C) of para-substituted benzene[4,5]	9
1164	ν(O-C(O)-O)[2,5]	2
1194	ν (C-CH_3) in gem-dimethyl position[6,7]	1
1230	ν(C-O-C) antisymmetric[8,9]	1
1364	δ(CH) of methyl groups[10]	
1505	ν $(C{=}C)_{Ar}$ [3,5]	3
1773	ν (C=O)[3,5]	3
2955	ν(CH)	13

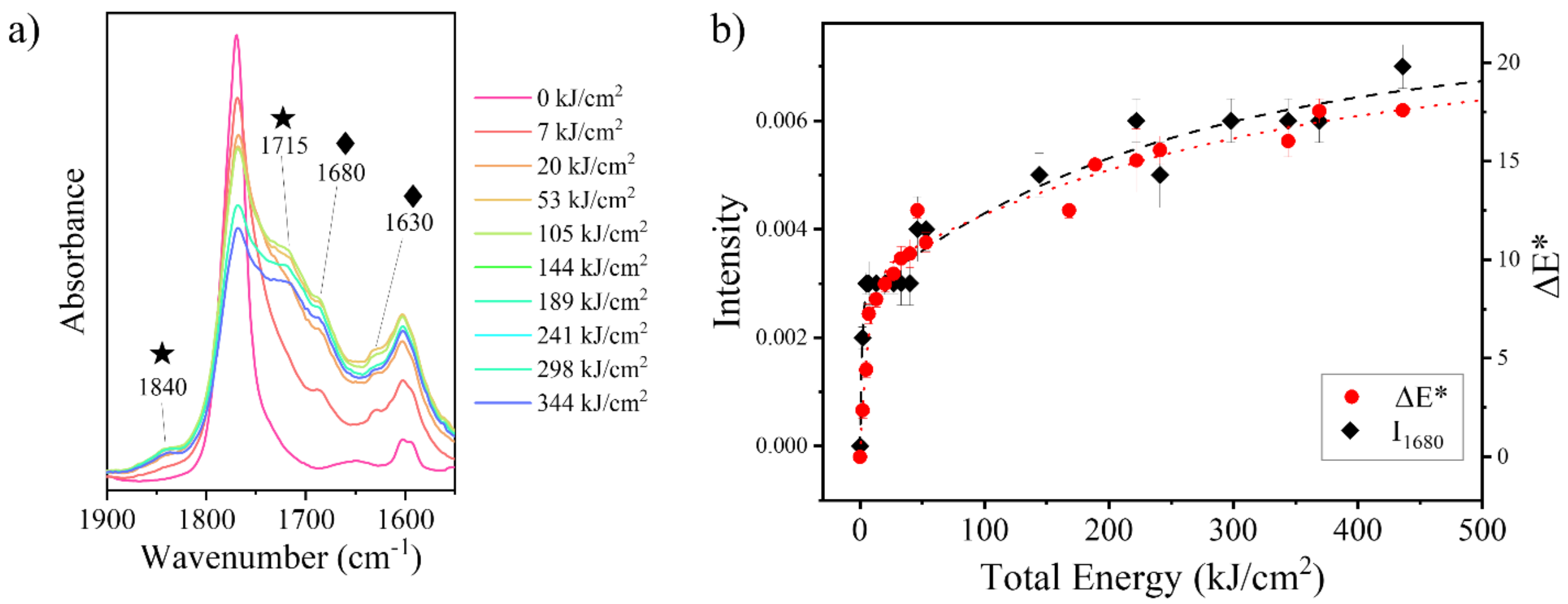

Figure S4: a) ATR spectra of polycarbonate samples in the carbonyl stretching range (1900-1600 cm^{-1}) for different total energies. In particular, bands at 1715 cm^{-1} and 1840 cm^{-1} associated with the photo-oxidation process and bands at 1680 cm^{-1} and 1630 cm^{-1} associated with the photo-Fries reaction are highlighted with ★ and ◆, respectively. b) ΔE* values and ATR intensity at 1680 cm^{-1} (Photo-Fries) as a function of total energy. The results of ATR measurements are comparable with those obtained with non-invasive external reflection IR spectroscopy.

Table S2: CIELAB color space coordinates ΔL*, Δa*, Δb*, and ΔE* and associated errors (δΔL, δΔa, δΔb and δΔE), calculated for the set of photochemically aged polycarbonate samples, under D65 standard illuminant and 10° standard observer conditions.

Total Energy (kJ/cm^2)	ΔL*	δΔL	Δa*	δΔa	Δb*	δΔb	ΔE*	δΔE
**2**	0.4	0.5	-1.0	0.1	2.1	0.3	2.4	0.4
**5**	-0.3	0.6	-1.5	0.2	4.2	0.4	4.4	0.5
**7**	-0.4	0.7	-2.4	0.2	6.9	0.5	7.3	0.6
**13**	-0.4	0.6	-2.8	0.2	7.5	0.4	8.0	0.4
**20**	-0.7	0.6	-2.9	0.2	8.3	0.3	8.8	0.4
**27**	-1.1	1.0	-3.0	0.3	8.7	0.4	9.3	0.6
**33**	-0.9	0.9	-3.4	0.2	9.5	0.5	10.0	0.6
**40**	-0.6	0.8	-3.4	0.2	9.7	0.7	10.3	0.7
**46**	-1.4	0.8	-3.7	0.1	11.9	0.3	12.5	0.4
**53**	-0.9	0.6	-3.6	0.2	10.2	0.4	10.9	0.5
**53**	-1.4	1.0	-3.1	0.2	11.1	0.5	11.6	0.7
**144**	-0.6	0.5	-3.4	0.1	9.7	0.3	10.3	0.3
**189**	-1.7	1.0	-3.8	0.2	14.2	0.3	14.8	0.5

**222**	-2.3	1.1	-3.4	0.8	14.5	1.3	15.0	1.6
**241**	-1.9	0.8	-3.8	0.4	15.0	0.6	15.6	0.7
**255**	-1.9	0.9	-4.3	0.1	16.7	0.3	17.3	0.4
**344**	-1.6	0.8	-3.8	0.4	15.5	0.7	16.0	0.8
**347**	-1.9	0.7	-4.4	0.2	16.9	0.5	17.5	0.6
**369**	-1.8	0.5	-4.3	0.2	16.9	0.4	17.6	0.5
**436**	-2.0	0.6	-4.0	0.2	17.0	0.2	17.6	0.3
**455**	-3.1	0.9	-3.9	0.4	16.4	0.6	17.1	0.8
**659**	-5.4	1.2	-3.6	0.2	18.7	0.4	19.8	0.8
**805**	-5.1	0.9	-3.4	0.2	17.6	0.5	18.6	0.8

Table S3: Fitting parameters for the intensity variation of the 1680 cm^{-1} band obtained from the external reflection spectra and from ΔE* values using exponential functions. The constants (d_1 and d_2), their associated errors, and weight percentages (W_{A1}% and W_{A2}%) are reported.

	d_1 (kJ/cm^2)	W_{A1}%	d_2 (kJ/cm^2)	W_{A2}%	Fitting equation
1680	4.6 ± 2.0	33	735 ± 120	67	$y = y_0 + A_1*exp(-x/d_1) + A_2*exp(-x/d_2)$
ΔE*	6.6 ± 1.5	45	310 ± 75	55	$y = y_0 + A_1*exp(-x/d_1) + A_2*exp(-x/d_2)$

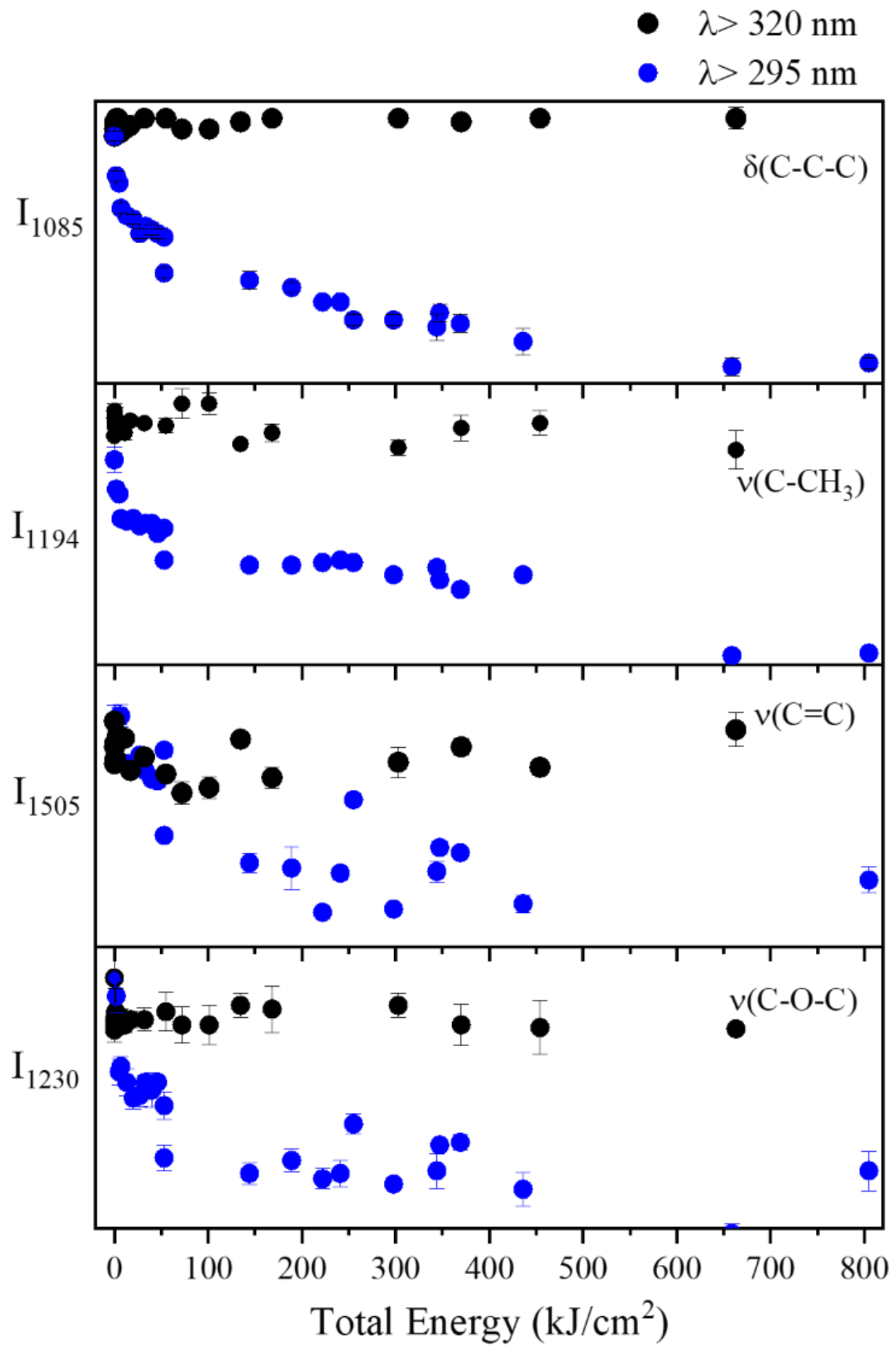

Figure S5: IR spectral variation of BPA-PC under outdoor (λ > 295nm, blue circles) and indoor (λ > 320 nm, black circles) light exposure (expressed as total energy) considering the following vibrational modes: δ(C-C-C) @ 1085 cm^{-1}, ν($C-CH_3$) @ 1194 cm^{-1}, ν(C=C) @ 1505 cm^{-1}, and ν(C-O-C) @ 1230 cm^{-1}. Error bars represent the standard deviation.

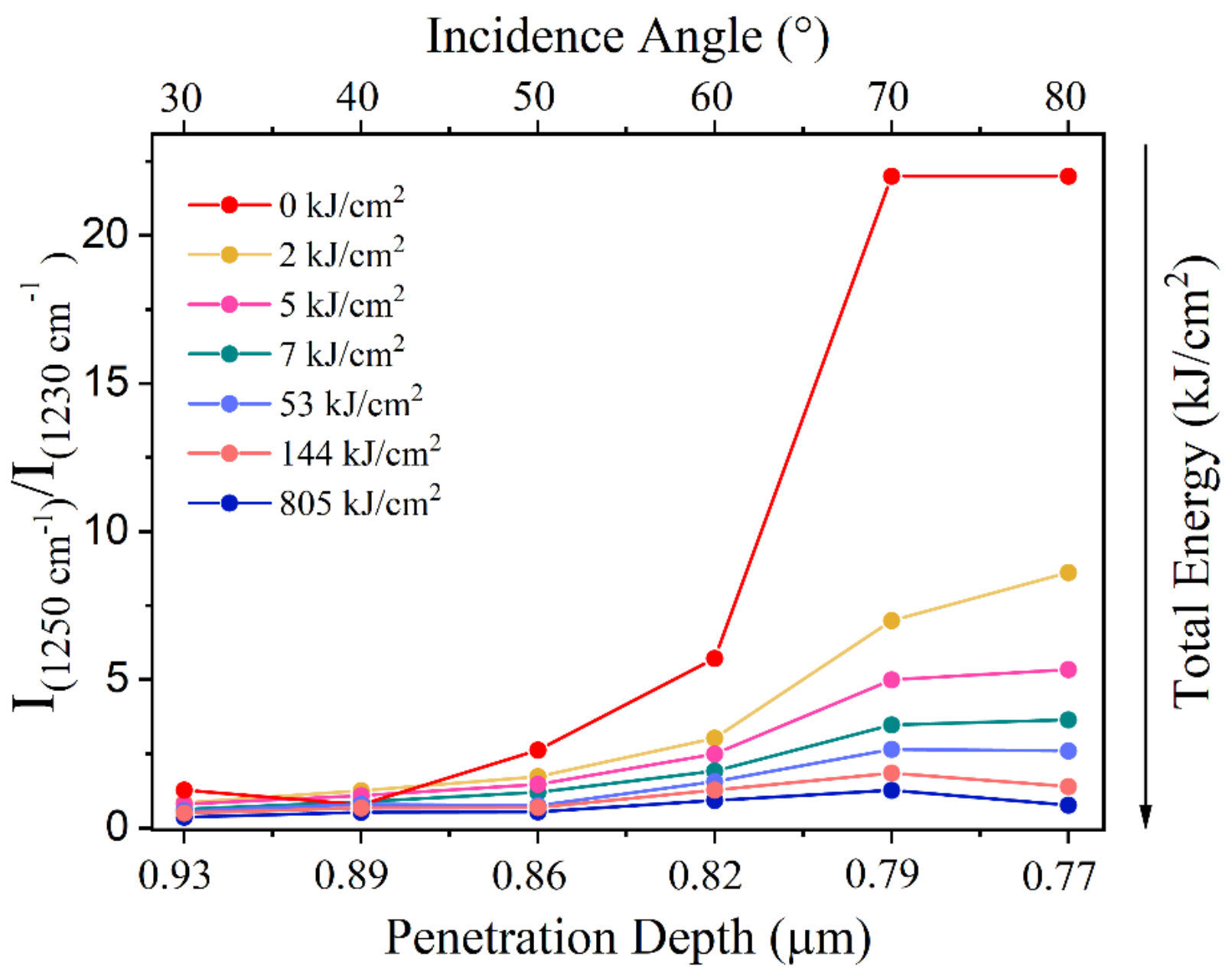

Figure S6: $I_{t\text{-}t}/I_{c\text{-}t}$ ratio attributed to the trans–trans and cis–trans configurations (at 1250 and 1225 cm^{-1}, respectively), observed under outdoor aging as a function of the total energy (kJ/cm²), penetration depth, and the corresponding angle of incidence on the material surface.

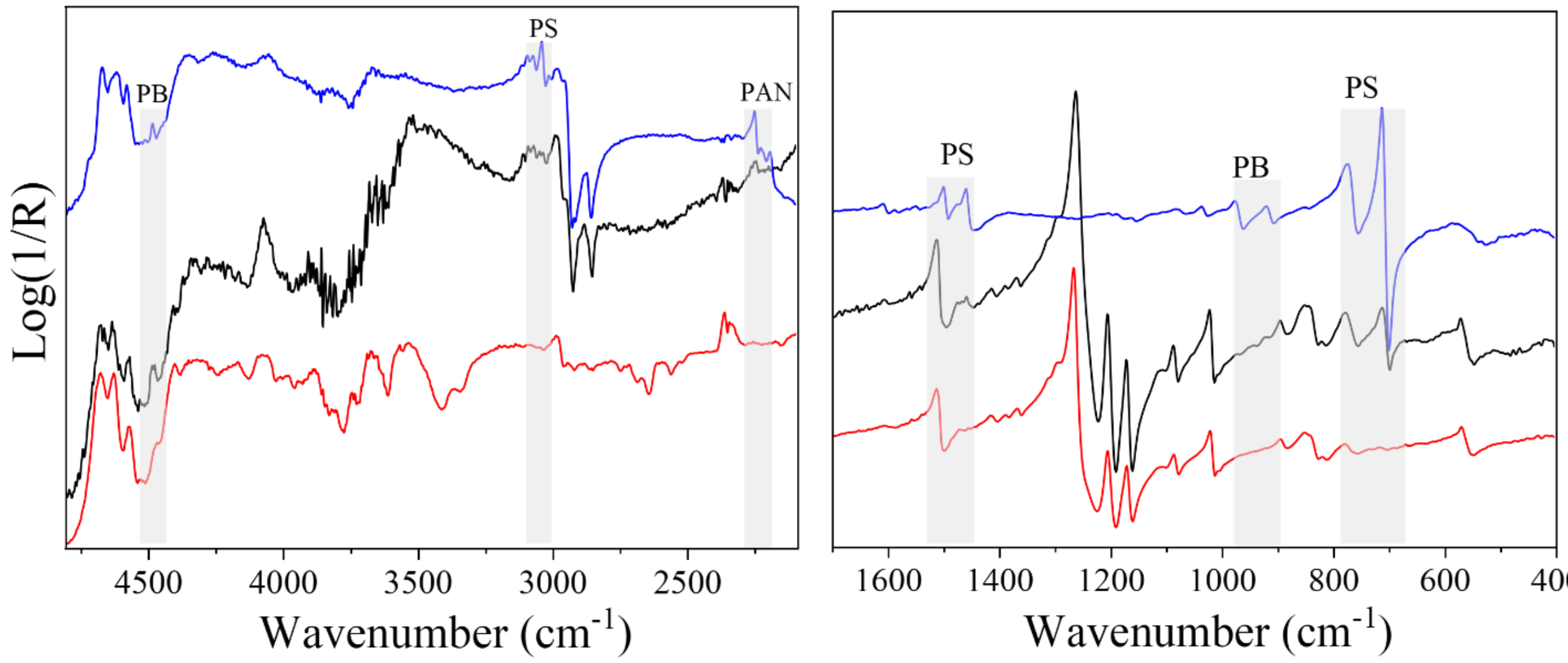

Figure S7: IR spectra of the Cellidor phone-0288 (black line) compared with reference spectra of BPA-PC (red line) and ABS (blue line) in the mid and near infrared range. Light grey rectangles mark the characteristic bands of the three monomer units constituting ABS PS= polystyrene; PB=polybutadiene; and PAN=polyacrylonitrile.

References

(1) Tan, C. Z. Determination of Surface Structure and the Depth Profile of Silica Glass by Infrared Spectroscopy. *Guangxue Jingmi GongchengOptics Precis. Eng.* **2005**, *13*, 413–420. https://doi.org/DOI:%252010.1016/s0022-3093(97)00438-9.
(2) Parshin, A. M.; Gunyakov, V. A.; Zyryanov, V. Y.; Shabanov, V. F. Domain Structures in Nematic Liquid Crystals on a Polycarbonate Surface. *Int. J. Mol. Sci.* **2013**, *14* (8), 16303–16320. https://doi.org/10.3390/ijms140816303.
(3) Kraus, R. G.; Emmons, E. D.; Thompson, J. S.; Covington, A. M. Infrared Absorption Spectroscopy of Polycarbonate at High Pressure. *J. Polym. Sci. Part B Polym. Phys.* **2008**, *46* (7), 734–742. https://doi.org/10.1002/polb.21405.
(4) Gu, X.; Qian, B.; Yu, L.; Wang, J.; Wang, Y. Mechanism of Infrared-Laser Engraving for Polycarbonate-Based Films. *Mater. Sci. Forum* **2020**, *1003*, 233–238. https://doi.org/10.4028/www.scientific.net/MSF.1003.233.
(5) Manfredi, M.; Barberis, E.; Marengo, E. Prediction and Classification of the Degradation State of Plastic Materials Used in Modern and Contemporary Art. *Appl. Phys. A* **2016**, *123* (1), 35–35. https://doi.org/10.1007/s00339-016-0663-x.
(6) Swinarew, A. S.; Swinarew, B.; Flak, T.; Okła, H.; Lenartowicz-Klik, M.; Barylski, A.; Popczyk, M.; Gabor, J.; Stanula, A. The Evaluation of Simulated Environmental Degradation of Polycarbonate Filled with Inorganic and Organic Reinforcements. *Polymers* **2021**, *13* (20). https://doi.org/10.3390/polym13203572.
(7) Collin, S.; Bussière, P. O.; Thérias, S.; Lambert, J. M.; Perdereau, J.; Gardette, J. L. Physicochemical and Mechanical Impacts of Photo-Ageing on Bisphenol a Polycarbonate. *Polym. Degrad. Stab.* **2012**, *97* (11), 2284–2293. https://doi.org/10.1016/J.POLYMDEGRADSTAB.2012.07.036.
(8) Heymans, N.; Van Rossum, S. FTIR Investigation of Structural Modifications during Low-Temperature Ageing of Polycarbonate. *J. Mater. Sci.* **2002**, *37*, 4273–4277. https://doi.org/10.1023/A:1020636115507.
(9) Heymans, N. FT i.r. Investigation of Structural Modification of Polycarbonate during Thermodynamical Treatments. *Polymer* **1997**, *38* (14), 3435–3440. https://doi.org/10.1016/S0032-3861(96)00929-9.
(10) Dehaye, F.; Balanzat, E.; Ferain, E.; Legras, R. Chemical Modifications Induced in Bisphenol A Polycarbonate by Swift Heavy Ions. *Nucl. Instrum. Methods Phys. Res. Sect. B Beam Interact. Mater. At.* **2003**, *209*, 103–112. https://doi.org/10.1016/S0168-583X(02)02048-7.
